# CADD v1.7: using protein language models, regulatory CNNs and other nucleotide-level scores to improve genome-wide variant predictions

**DOI:** 10.1093/nar/gkad989

**Published:** 2024-01-05

**Authors:** Max Schubach, Thorben Maass, Lusiné Nazaretyan, Sebastian Röner, Martin Kircher

**Affiliations:** Exploratory Diagnostic Sciences, Berlin Institute of Health at Charité – Universitätsmedizin Berlin, Berlin, Germany; Institute of Human Genetics, University Hospital Schleswig-Holstein, University of Lübeck, Lübeck, Germany; Exploratory Diagnostic Sciences, Berlin Institute of Health at Charité – Universitätsmedizin Berlin, Berlin, Germany; Exploratory Diagnostic Sciences, Berlin Institute of Health at Charité – Universitätsmedizin Berlin, Berlin, Germany; Exploratory Diagnostic Sciences, Berlin Institute of Health at Charité – Universitätsmedizin Berlin, Berlin, Germany; Institute of Human Genetics, University Hospital Schleswig-Holstein, University of Lübeck, Lübeck, Germany

## Abstract

Machine Learning-based scoring and classification of genetic variants aids the assessment of clinical findings and is employed to prioritize variants in diverse genetic studies and analyses. Combined Annotation-Dependent Depletion (CADD) is one of the first methods for the genome-wide prioritization of variants across different molecular functions and has been continuously developed and improved since its original publication. Here, we present our most recent release, CADD v1.7. We explored and integrated new annotation features, among them state-of-the-art protein language model scores (Meta ESM-1v), regulatory variant effect predictions (from sequence-based convolutional neural networks) and sequence conservation scores (Zoonomia). We evaluated the new version on data sets derived from ClinVar, ExAC/gnomAD and 1000 Genomes variants. For coding effects, we tested CADD on 31 Deep Mutational Scanning (DMS) data sets from ProteinGym and, for regulatory effect prediction, we used saturation mutagenesis reporter assay data of promoter and enhancer sequences. The inclusion of new features further improved the overall performance of CADD. As with previous releases, all data sets, genome-wide CADD v1.7 scores, scripts for on-site scoring and an easy-to-use webserver are readily provided via https://cadd.bihealth.org/ or https://cadd.gs.washington.edu/ to the community.

## Graphical Abstract

**Graphical Abstract:**
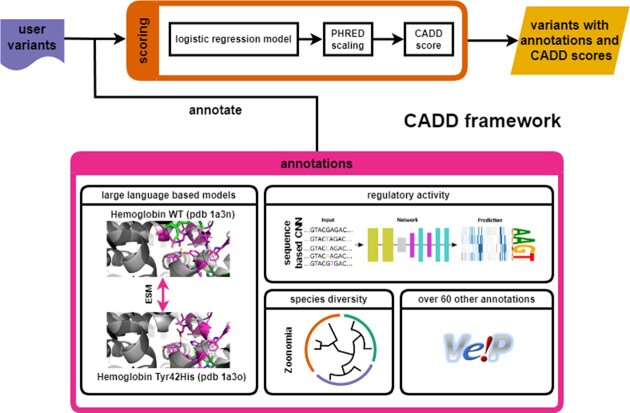

## Introduction

In recent years, the field of interpreting genetic variants has witnessed remarkable progress, driven by advancements in genomics, bioinformatics, and data analysis. The advent of high-throughput sequencing technologies has enabled researchers to generate vast amounts of genomic data, leading to an enhanced understanding of the role that genetic variants play in health and disease (1–3).

Despite significant progress, several challenges persist in accurately deciphering the complexities of the human genome. The vast number of genetic variants that are being identified makes it difficult to distinguish between neutral or benign variants and those that are clinically relevant or disease-causing. Additionally, many genetic variants exhibit subtle effects on gene function or disease susceptibility, requiring advanced computational methods and functional assays to uncover their potential impact. The genetic landscape is variable across diverse individuals and populations (i.e. haplotypes and frequencies), posing challenges in extrapolating findings about individual variants in certain patient groups (4–6). Furthermore, our understanding of the non-coding regions of the genome has evolved, revealing their crucial regulatory roles, but also adding complexity to variant interpretation (1,7–13). The lack of comprehensive functional readouts for many variants further complicates their interpretation (3,11,14–17). Finally, ethical and privacy concerns surrounding genetic data sharing and analysis must be carefully navigated (18,19).

Combined Annotation Dependent Depletion (CADD) is a Machine Learning-based scoring system used to predict the deleteriousness or functional impact of genetic variants in the human genome (20). It integrates various genomic annotations and functional information to assign a single numerical deleteriousness score to each variant, correlating with the likelihood that the variant is pathogenic or disruptive to gene function. CADD is based on logistic regression models and applicable to single nucleotide variants (SNVs) and short inserts and deletions (InDels), throughout the human genome reference assembly.

CADD makes use of a wide range of features, including DNA sequence properties, gene and transcript models, scoring of protein-coding effects, conservation across species, biochemical activity and other genomic annotations. By combining multiple sources of information, CADD aims to provide a more accurate prediction of variant deleteriousness compared to using individual features. Further, by integrating the partially correlated features in one score, it also overcomes issues from individually considering multiple annotations and presuming them as independent evidence for a variant effect. CADD has been widely adopted in the field of genomics and variant interpretation, aiding researchers and clinicians in prioritizing genetic variants for further analysis, especially in the context of disease-causing variants and personalized medicine applications (21–23). The principle behind CADD has been used to develop scores for further species (24–26), to infer measures of selection (27) and constraint (28) and extended to further variant classes, specifically structural variants (29). Further, the broader concept of using species differences and long-standing variation has been applied to develop improved models of missense effects (30,31).

**Figure 1.**
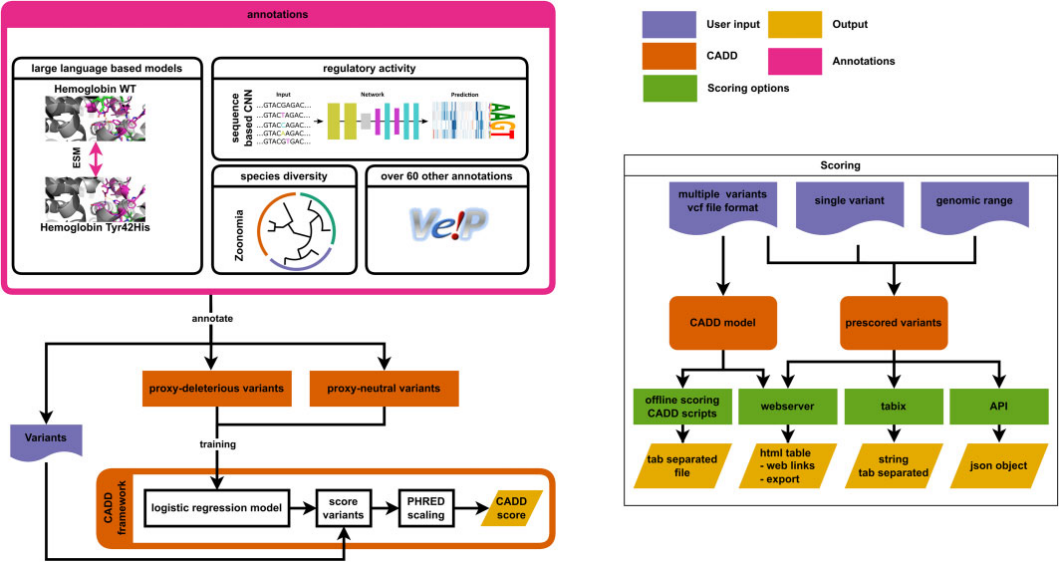
Combined Annotation Dependent Depletion (CADD) workflow. CADD is a Machine Learning-based scoring system for predicting the deleteriousness of genetic variants across the genome. It integrates various genomic annotations and functional information in a logistic regression model to assign numerical deleteriousness scores for all possible single nucleotide variants (SNVs), multi-nucleotide substitutions and insertion/deletion changes (InDels). From a user's perspective, a provided variant list (typically in Variant Call Format, VCF) is annotated with diverse annotation features. Combined CADD scores are determined and returned for the provided variants with or without the additional annotations. In CADD v1.7, we integrated new annotation features, among them state-of-the-art protein language model scores (Meta ESM-1v), regulatory variant effect predictions (from sequence-based convolutional neural networks) and sequence conservation scores (Zoonomia). Various sources are available for obtaining CADD scores. CADD models and offline-scoring are available to calculate and retrieve scores on-premises. A webserver offers the download of pre-scored SNV and InDel variants, a VCF upload for online-scoring and exploring pre-scored SNV variants directly on our website. Pre-scored variants are also accessible through HTSlib/tabix and API calls. PDB entries 1a3o and 1a3n were used to create graphical representations of Hemoglobin shown in the left-most panel.

As our understanding of genetics and genomics is rapidly evolving, CADD aims at continuous integration of new research discoveries to provide the most accurate predictions and to support the latest human genome builds. Since the CADD v1.6 release in 2021 (32), which specifically focused on improving the scoring of splice effects using sequence-based models, new methods for the identification of functional regions and the assessment of impact of variation in the human genome have been developed. In CADD version 1.7, we again added new features that improve CADD scores for certain variant effects (Figure 1), boosting the overall performance of CADD and bringing new developments to the community.

Specifically, deep learning methods that frequently outperform other models have been of continued interest to the community. Here, we include scores derived from the Evolutionary Scale Modeling (ESM) for assessment of variants in protein coding regions (33) as well as from a convolutional neural network (CNN) trained on open chromatin sequences, as a proxy for regulatory regions in the genome. Further, we complement the previously included conservation scores with the updates of the Zoonomia project (34) and include new annotations for 3′ Untranslated Regions (3′ UTRs) (35) as well as models of genome-wide mutational rate (36). Finally, we update our gene and transcript models by advancing from Ensembl version 95 to 110 and using an updated version of Ensembl Variant Effect Predictor (VEP). As for previous CADD versions, genome-wide scores, scripts for on-site scoring and an easy-to-use webserver are readily provided to the community for CADD v1.7 (Figure 1).

## Unique characteristics of the CADD framework

CADD is different from many other machine learning methods for variant prioritization that are frequently trained on the small number of genetic variants that have a known effect on health or disease (37–40). Instead, CADD uses a larger and less biased set of training data. It assumes that most of the variants that have appeared and stayed in humans after speciation are harmless or neutral, because they have survived millions of years of natural selection; we call these variants ‘proxy-neutral’. These variants are compared with a set of simulated variants that have not been purified by selection; while many of these variants are neutral, some of them would be harmful or have some effect if they occurred in a person; we call these variants ‘proxy-deleterious’. The main idea of CADD is to contrast the proxy-neutral and proxy-deleterious sets based on available genomic annotations using a machine learning approach, i.e. to learn that there are fewer harmful mutations in the proxy-neutral set by respective shifts in the annotation features. Thus, creating a model for estimating the deleteriousness of all types of short sequence variants, coding and non-coding, across the human genome.

The proxy-neutral set consists of more than 14 million human lineage derived sequence alterations (14 million SNVs and 1.7 million InDels up to 50 bp), inferred from primate whole genome alignments (41). The proxy-deleterious variation is a size matched set of simulated variants. We simulate these ‘*de novo*’ variants according to the substitution frequencies and insertion/deletion lengths observed in the proxy-neutral set, accommodating a local adjustment of mutation rates and asymmetric CpG-specific mutation rates (20). The resulting set of about 30 million variants is annotated with more than 100 annotations derived from gene model information, sequence conservation and constraint, epigenetic and regulatory activity, variant density, and others.

In all recent CADD implementations, we train a logistic regression model on this data. To account for some non-linear relation between annotations, CADD creates crossed feature annotations (20), like including conservation scores or epigenetic activity model coefficients based on the annotated consequence labels (e.g. missense, nonsense, upstream, downstream, intergenic). The resulting model is then applied genome-wide, i.e. we compute the logistic regression scores (raw scores) for all possible single base pair alterations of the human genome. Based on these genome-wide raw scores, a conversion table is generated that translates to a scaled CADD-score, a variants’ relative rank among all potential SNV alterations presented on PHRED-scale (−10 * log_10_(1/rank)). The conversion table is used to obtain scaled scores for any SNV, multi-nucleotide substitution or InDel change, representing the commonly used CADD scores (abbreviated as C-scores).

As raw scores are the immediate output from the machine learning model, they summarize the extent to which the variant is likely to have derived from the proxy-neutral (negative values) or proxy-deleterious (positive values) class. Raw scores have only relative meaning with higher values indicating that a variant is more likely to have derived from the proxy-deleterious than the proxy-neutral variant set. However, they change between distinct annotation combinations, training sets or tuning parameter choices (i.e. CADD model versions) and thus do not have absolute meaning and cannot be compared across models. Raw scores offer superior numerical resolution and preserve relative differences between scores that may otherwise be rounded away in the scaled *C*-scores. Thus, when statistically comparing score distributions between groups of variants (e.g. in cases versus controls), raw scores should be used (42).

In contrast, ‘PHRED-scaled’ C-scores are normalized to all potential ∼9 billion SNVs, and thereby can be compared across model versions and reference builds. Regardless of the annotation set or model parameters, a scaled score of 20 or greater indicates a raw score in the top 1% of all possible reference genome SNVs, and a score of 30 or greater indicates a raw score in the top 0.1%. When identifying causal variants or performing a fine-mapping of variants within associated loci, scaled scores are advantageous as they allow the user a direct interpretation in terms of the estimated deleteriousness relative to all possible SNVs in the reference genome.

Since its inception, we have been advocating for ranking variants by CADD scores rather than declaring a single universal cut-off value to declare a variant ‘pathogenic’ (or ‘functional’ or ‘deleterious’) as opposed to ‘benign’ (or ‘non-functional’ or ‘neutral’). We believe that such binarization is flawed due to its substantial loss of information and as the choice of the cut-off would naturally depend on specific factors, such as the severity of the phenotype and how much expression variation is tolerated (e.g. haploinsufficiency, dominance, recessiveness), or the amount of time and resources available for curation or experimental follow-up (42).

## Updates to the CADD annotation set

The CADD framework is highly flexible and modularized, enabling further improvements, like the integration of new annotations as the result of scientific developments. Despite its imperfect approximation of training set labels, the key advantage of the CADD framework is the comprehensive and systematic labeling of tens of millions of variants for the training set. Each iteration of the CADD model is therefore trained on about 30 million variants and hundreds of features derived from available annotations. This enables CADD to accommodate nearly any feature that can be tied to reference assembly coordinates, and the capacity to score both coding and non-coding variants. The size of the training set allows integration of many annotations without substantial risk of overfitting.

To explore potential updates to CADDs feature set, we reviewed recent literature for variant scores and annotations that could potentially improve the performance of CADD (Supplementary Table S1). Among these scores and annotations are APARENT2 (35), Zoonomia (34), Roulette (36) and gwRVIS scores (43). APARENT2 is the successor of APARENT, a sequence-based deep learning tool that quantifies a variants' potential to disrupt alternative polyadenylation (44). The Zoonomia project provides conservation information for each position in the genome by comparing the genomes of more than 200 mammalian species, substantially improving on CADD’s previously used 43 mammalian sequences (34). The increased number of species comes with higher resolution on the level of sequence conservation scores and more complete coverage along the genome. In previous versions, conservation has been a major indicator of which regions and nucleotides are prone to disease causing effects. It is also one of the most important predictors in the sparsely annotated non-coding genomic regions (45). Roulette is a sequence mutability score, i.e. a score reflecting the sequence and its context dependent propensity to acquire *de novo* mutations (36). GwRVIS is a genome-wide score that quantifies intolerance to variation with nucleotide resolution. Conservation information is however explicitly excluded, distinguishing it from phylogenetic scores (43).

We also derived scores from ESM-1v protein language models (33) for single amino acid substitutions, inframe insertions and deletions, as well as frameshifts and stop gains (see Supplementary Note S2 for more details). ESM-1v is a transformer protein language model developed by the Meta Fundamental AI Research Team that aims at predicting effects of missense variants. The 650 million parameter model has been trained directly on protein sequence databases in an unsupervised manner. It has been proposed that the model understands protein function on a molecular-to-atomic level and was shown by several groups to be one of the best performing tools for missense variant prediction (33,46,47).

In the field of predicting epigenetic properties of non-coding elements, significant progress was made by the development of deep learning architectures. Here, Enformer is the most prominent example as well as the most complex architecture with a wide sequence context around each central position (48). It was shown that Enformer can effectively predict epigenetic marks from DNA sequence alone and allows to infer the sequence motifs underlying functional sequence alterations. From these and other modeling efforts, open-chromatin information like DNase-seq or ATAC-seq seem to provide good approximations for predicting regulatory variant effects, for example the activity measured from massively parallel reporter assays (MPRAs) (48–51). However, due to Enformer's complexity, its many cell-type specific outputs, and its computational time requirements, it is not feasible for us to apply it genome-wide and to integrate it into CADD. Instead, we decided to train a small-scale convolutional neural network (CNN) model on open chromatin sequences from multiple cell-types (see Supplementary Note S3 for details). Depending on the benchmark MPRA data set, our model showed a performance comparable to Enformer with much faster calculation times, motivating us to test its integration into CADD.

To evaluate whether the new annotations improve CADD’s performance, we collected several suitable benchmark data sets (see Supplementary Note S1). Our previous CADD model (CADD v1.6) served to determine a baseline performance on the benchmark sets and was compared with CADD models with one of the new annotations added (potentially resulting in multiple new CADD model features being incorporated). Further, we included the respective annotations or features as a standalone score in our benchmarks. We considered a significant improvement of the individual annotation added to CADD over the baseline model as sufficient criterion for integrating the respective score or annotation in our new CADD v1.7 model, which then included all new features.

## Protein language models

**Figure 2.**
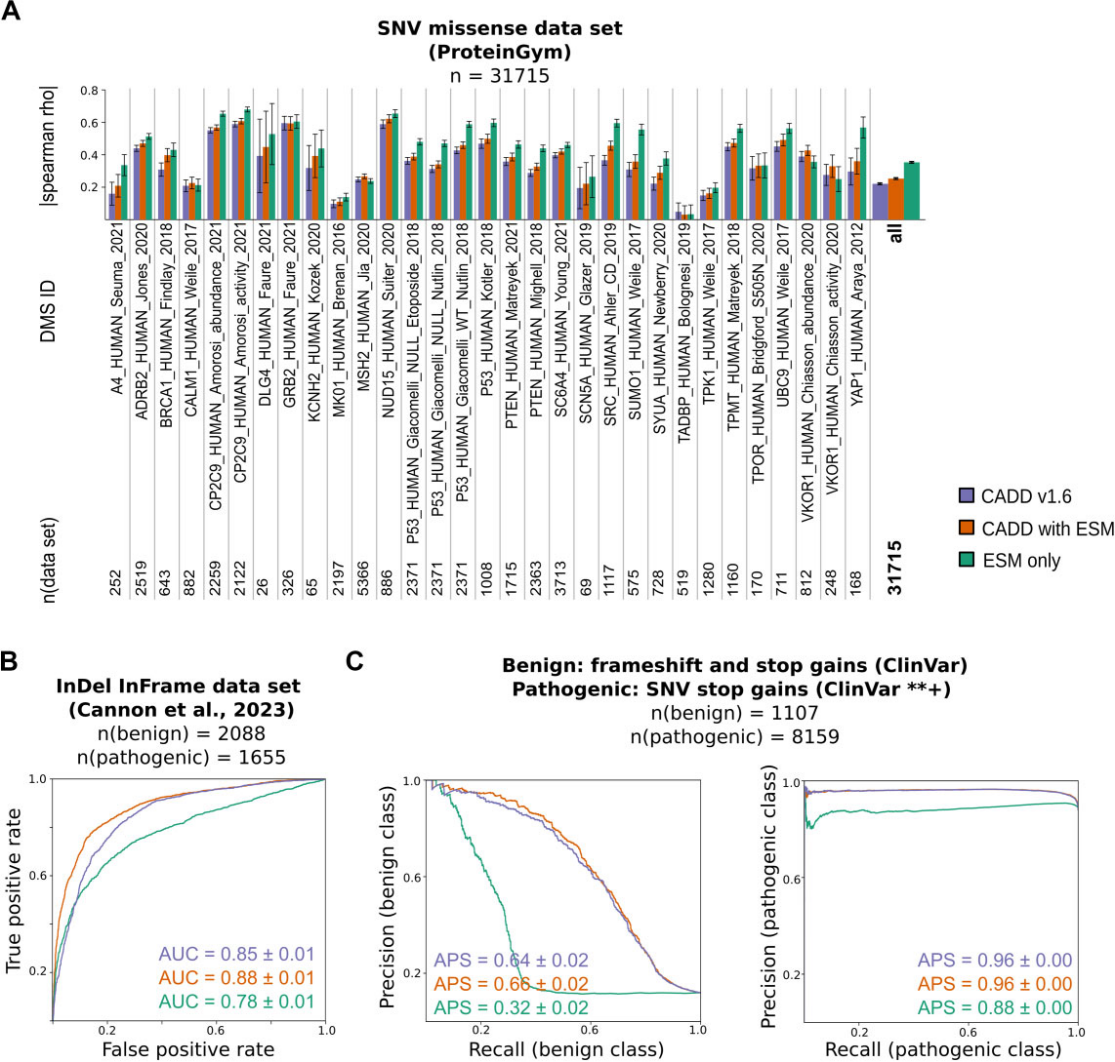
Including ESM protein language model scores improves the performance of CADD in coding regions. (**A**) Spearman correlations of CADD v1.6, a CADD model including ESM protein language model scores for missense variants, and the stand-alone ESM protein language model score for missense variants with experimental effect scores form the ProteinGym (52). (**B**,**C**) Receiver operating characteristics (ROC) curves and precision recall curves with the corresponding area under the ROC (AUC) and average precision score (APS) values. These metrics are shown for CADD v1.6, a CADD model including our ESM score for inframe InDel variants (B), for stop gain or frameshift variants (C), and for the derived ESM scores as stand-alone predictors (B, C). See also Supplementary Figure S2a for ROC curves of the data set shown in (C). The positive class corresponds to the pathogenic class if not stated otherwise.

The ESM-v1 language model for protein coding sequences consists of five models (initiated with different seeds during training) whose outputs are typically averaged. Provided with only an amino acid (AA) sequence, the models return likelihoods for specific AAs at each position along the sequence. To score missense variants, we used the average log odds ratio between the alternative and reference amino acid across the ESM-v1 models in a window of 350 AAs (see Supplementary Note S2 for details). To test the impact of the ESM-1v-derived missense score, we used Deep Mutational Scanning (DMS) data sets of human proteins from the ProteinGym database (52). We considered all amino acid substitutions that can occur due to a change of a single base pair and obtained about 41 000 DMS scores corresponding to about 31 000 variants (see Supplementary Notes S1 and S6). Next, we correlated DMS scores with CADD scores (Figure 2A). By including the ESM-1v-derived missense score in our CADD model, we observe an improvement for 29 of the 31 data sets and an overall improvement from the Spearman correlation of 0.217 ± 0.006 for the CADD v1.6 baseline model, to 0.250 +/- 0.006 for the model with ESM (Figure 2A). Of note, experimental DMS scores and our ESM-derived scores provide information on the effect of a variant on the protein level only, excluding for example splice effects. The extended CADD model does not reach the performance of the ESM-1v-derived missense score on these DMS effects. However, testing the scores on clinically relevant variants from ClinVar with two and more star reviewer status (abbreviated as **+; see Supplementary Note S1 and S6) shows that CADD outperforms ESM-1v-derived missense scores (Supplementary Figure S1a), when multiple molecular effects integrated in CADD can contribute to a variants' deleteriousness.

For the ESM-1v-derived score for inframe insertions and deletions (modeled as the log odds ratio of reference vs. alternative sequence in a context of 250 AAs, see Supplementary Note S2 for details), we used variants from two previously collected test data sets (53). In brief, the first data set contains benign and pathogenic variants from gnomAD v3.1 (54), ClinVar (37), and the Deciphering Developmental Disorder (DDD) study (55), resulting in a total of 2088 benign and 1655 pathogenic variants (see also Supplementary Notes S1 and S6). We observe an increase in area under the receiver operating characteristic curve (AUROC) values from 0.85 to 0.88 for our CADD model containing the ESM-1v-derived inframe insertion and deletion scores (Figure 2b). Here, the ESM-1v-derived score as a stand-alone predictor does not reach the performance of our CADD models. The second data set is a subset containing only variants from the DDD study (80 benign and 70 pathogenic variants; see also Supplementary Notes S1 and S6). We also report a positive trend for this data set, but note that the uncertainty associated with AUROC values is relatively large due to the small number of variants (Supplementary Figure S1b).

We also derived an ESM-1v score for frameshifts and stop gains by considering the median effects of lost amino acids in the log ratio (see Supplementary Note S2 for details). However, validation of the score proved difficult as we were not able to identify a suitable set of benign frameshift and stop gain variants to assess the performance. Pathogenic variants (**+ ClinVar reviewer status) were readily obtained using ClinVar InDels resulting in frameshifts (11 574 variants, see Supplementary Note S1 and S6) or SNVs resulting in stop gains (8159 variants, see Supplementary Note S1 and S6). In generating a benign variant set from ClinVar, we identified only 3 stop gains from InDels <50 bp, 15 stop gains from SNVs, as well as 44 frameshifts (see Supplementary Note S1). Testing our score on these data sets showed a positive trend but no significant improvement of the corresponding AUROC values when including the ESM-1v-derived scores (Supplementary Figure S2). Using ClinVar stop gains and frameshifts, independent of their review status for the benign set, gave us a total number of 1107 variants (see Supplementary Notes S1 and S6), which together with the pathogenic ClinVar **+ variants resulted in a more balanced test data set and confirmed the positive trend (Figure 2C). Of note, we also tried to utilize gnomAD v3.1 to obtain benign variants but filtering for frameshift and stop gain variants with an allele frequency above 0.5% resulted in less than 400 variants. In summary, the applied validation sets did not result in a measurable performance gain for frameshifts and stop gains, but we still included the ESM-derived scores for these variant classes in CADD v1.7 based on the positive trend.

## Regulatory sequence models

We trained a CNN model that predicts probabilities for variants being part of a regulatory element. The model was trained on DNase-seq data of seven different well studied ENCODE cell lines (see Supplementary Note S3, Figures S3 and S4, Table S2 for more information) and GC-matched background sequences as negative sample to improve the multitask performance of the model (56). The structure and parameters of the deep neural net were optimized, resulting in the final model (called RegSeq) which uses 500 bp of input sequence centered at the variant and has three convolutional layers and two dense layers (see Supplementary Table S3 for hyperparameter optimization and Supplementary Figure S5 for a schematic model overview).

We benchmarked our model with experimental reporter assay regulatory variant activity readouts from saturation mutagenesis MPRAs of different promoter and enhancer elements (57) (see Supplementary Note S3, Supplementary Figures S6 and S7). RegSeq showed an improved prediction over CADD v1.6 using cell-type specific, agnostic (average over 7 cell lines), and GC-matched background variant effect predictions. Pearson and Spearman correlations were similar (Supplementary Figures S6 and S7) to the performance of the larger Enformer model (48), which is based on a transformer architecture and more than 100 kb sequence context. Enformer is currently one of the best performing tools on variant effect prediction for regulatory sequence effects (48,58,59) and it would have been our preferred target for integration in CADD. However, the slow prediction time per variant effect (around 4 s per variant on one GPU) made it unreasonable to score the CADD training data and nearly impossible to create genome-wide predictions, motivating our decision to build and integrate the considerably smaller model (RegSeq).

**Figure 3.**
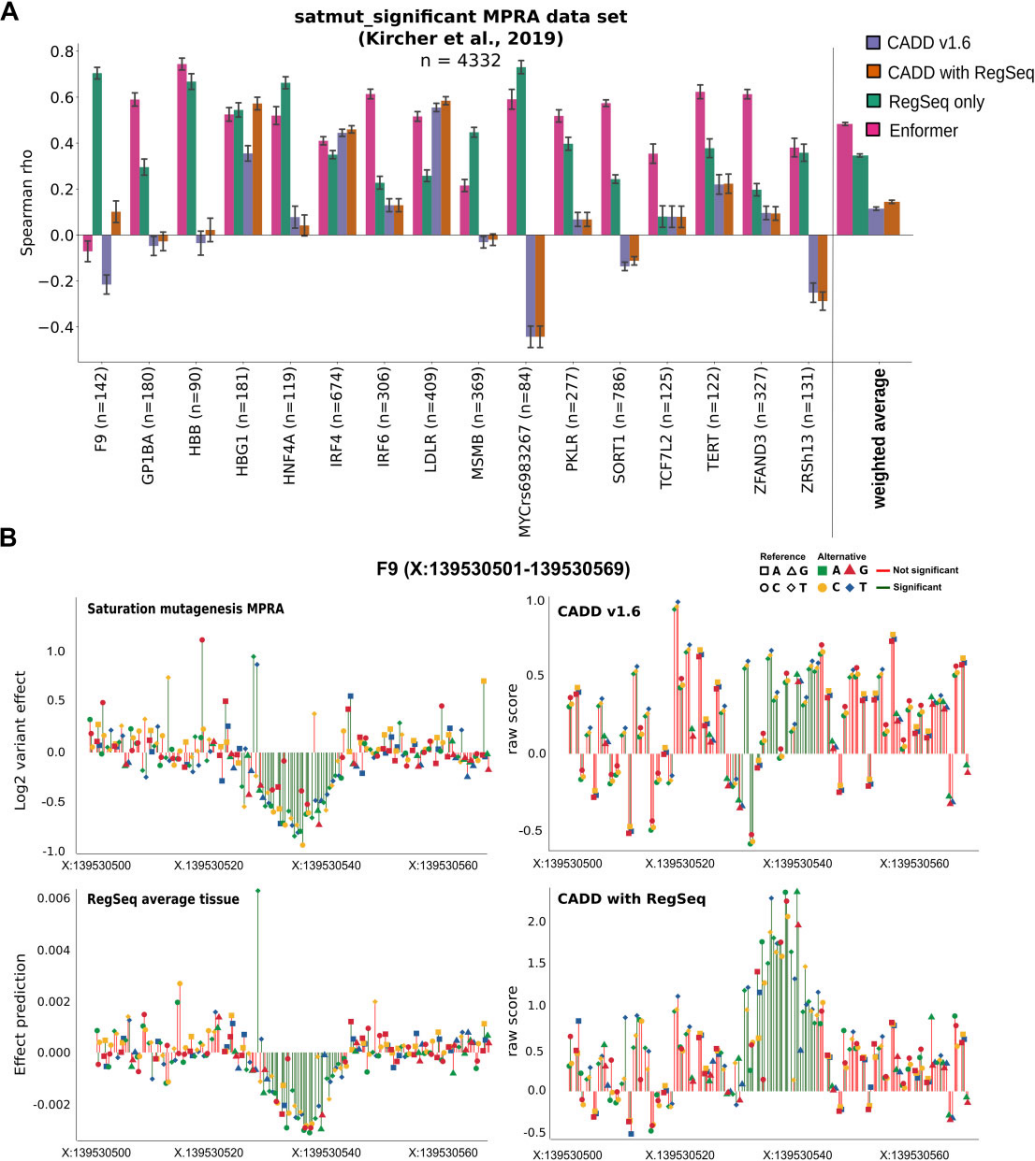
Integration of RegSeq features within CADD. Panel (**A**) shows the Spearman correlation of significant saturation mutagenesis reporter readouts (1 bp deletions and SNVs with min. 10 barcodes and *P*-value < 10^−5^) per element (57) with CADD v1.6 with and without integrated RegSeq features, an individual RegSeq feature (average across cell-types) and Enformer's average DNase-seq predictions (48). The absolute value of the MPRA effect is used for CADD, as it is not expected to predict the directionality of the expression effect. Weighted average is the mean Spearman correlation weighted by the number of variants in each element. Bars show the average correlation after bootstrapping (1000 runs with 80% of element variants) and error bars represent the standard deviation. Other data sets and Pearson correlation values are available in Supplementary Figures S8 and S9. Panel (**B**) shows part of the *Factor IX (F9)* promoter, where mutations lead to a significant reduction in activity due to association with *ETS*-related factors (57). The RegSeq model (lower left) aligns well with the variant effects measured by MPRA (upper left). CADD v1.6 with integrated RegSeq features aligns better with the motif (lower right) compared to the original CADD v1.6 (upper right). The whole *F9* promoter region is shown in Supplementary Figure S10.

We integrated the cell-type agnostic (average) RegSeq variant effect prediction as well as the GC-matched background predictions in CADD using several transformations on positive (activating) and negative (repressive) output predictions (e.g. minimum and maximum over cell-types; see Supplementary Note S3 and Supplementary Table S4). We trained a new CADD model with the additional RegSeq features and correlated the output with the significant experimental effects from the saturation mutagenesis data (Supplementary Note S1). Figure 3A shows the Spearman correlation of all variants (1 bp deletions and SNVs, *n* = 4332) per MPRA element before and after integration of RegSeq features (see correlation of all variants across elements in Supplementary Figure S8 and S9). We see an improved performance over nearly all elements as well as an overall improvement over the regulatory variant effects. Figure 3B shows a subpart of the promoter of the gene *Factor IX (F9)* with a binding site of *ETS*-related factors. The RegSeq model as well as the extended CADD v1.6 model with its integration can recover the binding site. We conclude that new sequence-based regulatory features are effectively used in the logistic regression model and result in improved regulatory variant effect predictions.

## 3′UTR, non-coding constraint, mutational scores and conservation scores

**Figure 4.**
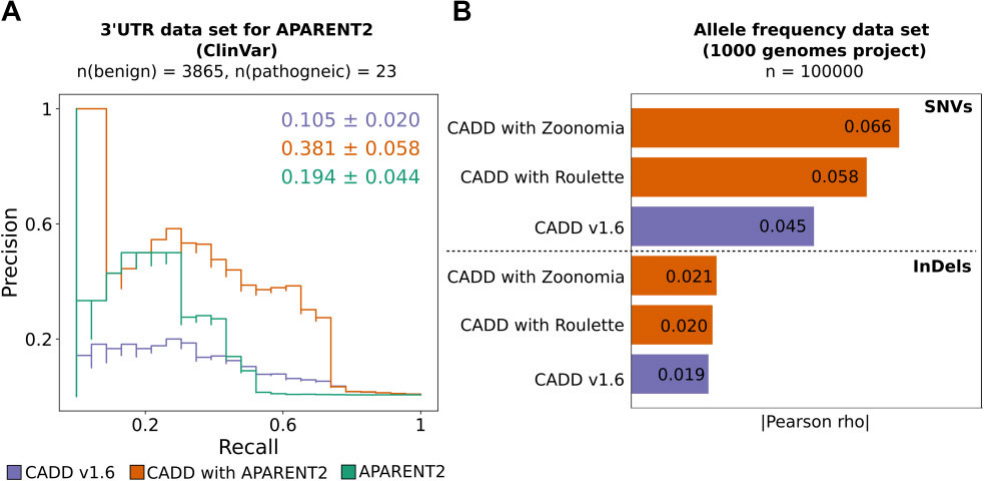
Comparison of CADD performance with and without APARENT2, Roulette, and Zoonomia features. (**A**) Precision recall curves and corresponding Average Precision Scores (APS) values of CADD v1.6, CADD including APARENT2 (35) and the absolute value of the APARENT2 score. The positive class corresponds to the pathogenic class. (**B**) Pearson correlation coefficients of CADD scores from models with and without Roulette (36) or Zoonomia (34) features with minor allele frequencies of 100 000 SNV and InDel variants sampled from the 1000 Genomes Project (64).

We and others have observed that CADD scores have good performance in coding regions of the genome whereas deleterious non-coding variants are more difficult to analyze with it (60–63). We believe that this is mostly due to a limited number of predictors of molecular effects outside of coding sequence (13,17,32). To improve CADD’s performance, new annotations targeted at non-coding variants were explored. First, we considered a sequence-based deep-learning score, APARENT2, quantifying the impact of genetic variation on transcript polyadenylation and 3′ cleavage (35). To assess the performance of CADD models with and without APARENT2, we tested them on pathogenic and benign 3′UTR variants from ClinVar. We only used variants for which a precalculated APARENT2 score was available (i.e. 23 pathogenic and 3865 benign variants, see also Supplementary Notes S1 and S6). We observe a significant improvement of average precision scores (APS) from 0.105 to 0.381 (Figure 4A). However, due to the small set of pathogenic variants, the uncertainty is rather large (standard deviation of 0.06).

Next, the genome-wide residual variation intolerance score (gwRVIS) was tested as an additional non-coding feature (43). It models intolerance to variation from functional genomic annotations and primary genomic sequence to prioritize non-coding variants. Notably, the score does not use conservation information, which, its authors argue, should better reflect human-specific constraint regions. To assess the impact of integrating gwRVIS in CADD, we used 3′UTR and 5′UTR variants as well as non-coding transcripts and intergenic variants from ClinVar for which a gwRVIS score was available (see also Supplementary Notes S1 and S6). We observe little predictive power of gwRVIS on this evaluation data set and a negative impact on CADD when adding this score into the feature set (Supplementary Figure S11), which is why we did not include gwRVIS in the new CADD release.

We further investigated the impact of the genome-wide mutation score Roulette (36). It models local mutation rate along the genome by including known determinants like the extended sequence context of 6 upstream and 6 downstream nucleotides, methylation, expression, transcription and replication directions and the observed mutability of each tri-nucleotide context in 50 kb windows. To assess the impact of Roulette, we needed a genome-wide measure of variant constraint and used variant minor allele frequency as a proxy. We therefore calculate the Pearson correlation coefficients of the respective CADD models with minor allele frequencies derived for a random subset of SNV and InDel variants (*n* = 100 000 each) in the 1000 Genomes project (64). We observe an increase in the absolute value of the correlation coefficients, especially for SNVs from 0.045 to 0.058 when including Roulette (Figure 4b).

As outlined above, we also explored the impact of the higher depth Zoonomia alignments and included metrics derived from the 43 primate genome and 241 mammalian genome alignments (34). This included phyloP and phastCons conservation scores available at base-level resolution, a set of annotated Ultra Conserved Elements (UCE, at least 235 species aligned and all aligning species are fixed for the same base at every position) and a set of Runs of Contiguous Constraint (RoCC, genomic regions where contiguous bases have phyloP score >2.27 in the mammalian alignment). When tested on correlation with minor allele frequencies as described for Roulette above, we observe an increase for SNVs from 0.045 to 0.066 when including Zoonomia conservation scores (Figure 4b).

## An updated CADD model

After validating the impact of the above-presented individual features, we combined them and added them to the features already included in CADD v1.6. We further updated the Ensembl transcript database as well as the Ensembl VEP tool (65,66) from version 95 to the most recent version 110 and trained a new CADD model (CADD v1.7). The model was tested and compared to CADD v1.6 on all ClinVar variants annotated with either benign (*n* = 35 037) or pathogenic (*n* = 26 981) clinical assertion with a two star and better reviewer status. In an additional assessment, we substituted the benign variants with variants from ExAC with a minor allele frequency ≥5% (*n* = 69 543; see Supplementary Note S1 and S6). On this set of variants, predominantly in coding genes, we see slight but significant improvements in AUROC values from 0.981 to 0.982 or from 0.985 to 0.986 (Supplementary Figure S12), respectively. Even though we are aware that variants with clinical assertions in the ClinVar database come with their issues and reflect by no means the entire genome (13,67), we think that this test unambiguously shows the improvement of CADD with the incorporation of new annotations.

CADD has been compared in many benchmarks and to many other tools in the past (32,46,47), which is why we are not presenting an extensive benchmarking here. Benchmarks are frequently limited to certain molecular functions or also use variants with clinical assertions, which brings up another frequently encountered limitation, namely that some tools are explicitly trained using ClinVar or similar variant sets. This makes it impossible to assess their performance on clinically relevant variants in an unbiased way, as there are no independent data sets that would not share the historic ascertainment or other basic characteristics of the ClinVar database. It should also be noted that distinguishing methods by their means of training only (e.g. supervised vs. unsupervised learning approaches) is insufficient and that the training objective needs to be considered (46,47). The inclusion of features derived from training on pathogenic variants may not immediately render a method unsuitable for comparison but may just complicate its benchmarking, as variants used in training the included feature may need to be eventually excluded. However, CADD integrates PolyPhen missense scores as a feature, a method that was trained on (a comparably small number by current standards) known pathogenic variants, while CADD’s training objective is the separation of human-derived from simulated variants. CADD’s initial publication (20) therefore included a benchmark of a model with and without PolyPhen scores, showing that CADD’s performance is not tainted by the inclusion of PolyPhen scores.

Another challenge of these benchmarks is that ClinVar and other clinically ascertained variant sources remain largely constrained to variants in and around protein-coding variants, despite the increased application of genome sequencing over the last few years (13). This is caused by our still limited understanding of variants in regulatory sequences and the large computational and experimental burden of analyzing the other approximately 98% of the genome (16,68). Additionally, specialized tools may perform better on specific benchmarking tasks, while scores like CADD integrate across molecular functions and provide genome-wide predictions (32). This makes fair comparisons difficult, as shown by the analysis of CADD and ESM-1v scores above. When comparing the two tools on protein function specific experimental assays (e.g. DMS data; Figure 2A), ESM-1v clearly outperforms CADD. ESM was designed to learn basic principles of protein sequence to structure and function relations, without the specific genomic encoding involving splicing, codon usage or for example mutational processes. Thus, on a set of missense variants that may have effects along genomic encoding, transcription, translation and protein function (e.g. ClinVar missense SNVs, Figure S1a), CADD outperforms ESM-1v. For such a data set, CADD profits from the many genomic annotations and features that contribute to variant deleteriousness.

## Web access and score availability

CADD and its associated software are freely available for all non-commercial applications and otherwise currently licensed through the University of Washington, Seattle. CADD scores are available for SNVs as well as InDels shorter than 50 bp located on the 22 human autosomes and chromosome X. We further provide scores for chromosome Y, although not all model annotations are available. Due to a lack of available annotations and consistent consideration across various studies, we do not support alternative haplotypes and other contigs. Due to differences in inheritance, gene density, transcription machinery and the availability of annotations, we no longer support scoring of mitochondrial variants in CADD v1.4 and later versions. While CADD will return scores for variants 50 bp and longer, this is outside of the range that it was trained on and scoring should be done with dedicated tools like CADD-SV (29).

CADD scores can be accessed in various ways, including through a number of third-party sources, such as dbNSFP (69), as a plug-in for Ensembl VEP (66), ANNOVAR (70), SeattleSeq (71), ExAC/gnomAD (54,72) and PopViz (73), but we recommend users to access information, scripts and pre-scored files primarily from our US (https://cadd.gs.washington.edu/) or Germany-based (https://cadd.bihealth.org/) webservers (see also Figure 1). These servers allow retrieval of SNV scores by position or in ranges, on the website or through a REST-API. In order to enable external sources to refer directly to CADD scores on our webservers, we implemented direct and versioned links to the scores of SNVs (e.g. https://CADD-SERVER/snv/BUILD-VERSION_inclAnno/CHROM:POS_REF_ALT). Genome-wide pre-scored SNV files as well as select pre-scored InDels are available for download. Additionally, VCF files with up to 100 000 positions can be uploaded and annotated with CADD scores, and users are informed by email once the processing is finished and variants can be downloaded. All scripts and annotations required for scoring SNVs and InDels are linked and available in the public GitHub repository (https://github.com/kircherlab/CADD-scripts).

We provide software for offline scoring especially for users that are legally required to score SNV and InDel variants on their own systems (18,74). Offline scoring takes a VCF file as input and allows for retrieval of annotations from pre-scored variant sets (to reduce computational time), as well as direct annotation and scoring of the remaining variants. It returns a gzip-compressed tab-separated text file (tsv.gz) containing all scored variants, with or without annotations. Offline scoring is based on the workflow management system Snakemake (75) with dependency management through conda (https://conda.io). We provide an installation script that downloads all necessary annotations and, optionally, pre-scored variants.

Further, we provide bigWig files of the maximum SNV score per genomic position that can be visualized as browser tracks for utilities like the UCSC genome browser or Integrative Genomics Viewer (IGV) and allow users to screen larger genomic areas quickly. Finally, the proxy-benign and proxy-pathogenic variant sets, the annotated training data as well as a comprehensive set of test and validation sets are available for other research efforts (https://cadd.gs.washington.edu/training or https://cadd.bihealth.org/training).

## Discussion and future work

We present a new release of the Combined Annotation Dependent Depletion scores (CADD version 1.7) that integrates annotations from recent community efforts on the assessment of variant effects, as well as new conservation and mutation scores. Over recent years, various deep learning methods have shown their potential to outperform other kinds of models and we include deep learning scores derived from Evolutionary Scale Modeling in protein coding regions (33) as well as a CNN model of open chromatin regions for the effects in regulatory regions. Further, we included conservation scores with five times more species from the Zoonomia project (34), a new annotation for 3′UTRs (35), and models of genome-wide mutational rates (36). Finally, we updated our gene and transcript models and version of Ensembl VEP (65,66).

For about a decade, CADD has been one of few machine learning methods able to provide genome-wide scores for SNVs, multi-nucleotide substitutions and InDels. Even though most known variants with a clinically relevant effect are in coding regions, it is probably the non-coding part of the genome that is key to delivering diagnoses to the many undiagnosed patients as well as understanding gene expression and regulation of all molecular processes of the cell. This is also not a new realization. For example in 1975, it was described that protein-coding differences between humans and chimpanzees were insufficient to explain their phenotypic differences (76). A recent study substantiated this and found only 126 out of 24 374 human-specific variants that are coding missense variants (77). Thus, we must presume that future versions of CADD will continue to put efforts in improving the deleterious predictions of non-coding variant effects.

While we show that integrating carefully selected annotations into CADD can improve its performance to predict deleteriousness, the model complexity (and computational expenses) increases with each new annotation. Especially deep neural network derived annotations, like the large language model ESM, are optimized for GPUs and the respective infrastructure is required to perform predictions on a genome-wide scale. We have taken up much of this computational burden by pre-calculating scores for all potential SNVs, but for users it still complicates running CADD scoring on their own infrastructure, for example when calculating scores of multi-nucleotide substitutions or new InDels offline. In such cases, the sequence models can also be run on regular CPUs, however with substantial performance loss. While ESM derived scores are only calculated for coding parts of the genome, regulatory effects need to be scored for approximately 50x more genomic positions. Thus, we already implemented a simpler network for regulatory variants, so that we were able to build the new CADD model, pre-score variants and enable scoring of variants in a reasonable amount of time on CPUs. Consequently, there is a need for simpler models, that achieve good but not necessarily the best performance for genome-wide scoring purposes, and we hope to raise awareness within the scientific community. We note that this also has an environmental impact.

As an organismal and genome-wide model of variant effects, CADD cannot represent the significance of individual genes for specific diseases (78,79). Existing gene and transcript specific information may therefore aid variant prioritization, independent and in addition to the ranking of variants by CADD scores. For example, information about the specific phenotype (including pathways, gene interactions, or affected tissues) is potentially of high relevance. This may motivate the integration of gene or transcript level annotations into genome-wide models like CADD. However, if we use measures that are specific to each gene and transcript, such as essentiality, protein interactions and network centrality, or expression specificity, this could in principle also impair the discovery of less well-studied disease genes due to observation biases (80). Further, to combine annotations into genome-wide models, they should be at nucleotide resolution, available for all variants in a class, and without major biases. Thus, even though other information is useful for a final variant ranking, we are currently skeptical of integrating broad-resolution annotations that prioritize variants based on their location in specific genomic regions.

Another extension of CADD that we have been considering is the extension, or better described as a split-out, to cell-type specific models. Tens of thousands of functional genomics data sets are available, for example through the NBCI Gene Expression Omnibus (GEO) (81)—including gene expression, DNA accessibility, immunoprecipitation of DNA binding (transcription factors and histones), DNA methylation, 3D organization and interaction of DNA elements (e.g. enhancer-promoter links) for various cell-types, whole tissues or single cell experiments. The ENCODE project and others initiated data portals with versioned and uniform data processing pipelines as well as explored imputation of molecular assays resulting from such data (3,82). Further, the availability of single cell atlas data has substantially increased over the last years. While an individual integration is clearly not feasible, it can be speculated that cell-type specific sequence model representation of these data sets could reduce the sheer number of data sets and has the potential to remove individual biases by combining many experiments. However, cell-types are not equidistant in expression and molecular profiles. Thus, without a trajectory or ‘phylogenetic’ tree of cell-types, the result would be many correlated CADD models for some selection of cell-types. It is therefore critical to first describe the relationship between cell-types and to integrate data across a continuous Waddington-like landscape of cell-types and cell states (83). Such a model would be the goal for bringing cell-type effects to the challenge of identifying disease or phenotype causal variants on the organismal level.

On an unrelated note, CADD v1.7 might be the last version to support the human reference genome sequence GRCh37, a version that was succeeded by the better and more complete GRCh38 version in December 2013. In the future, we want to focus on the support of GRCh38 as well as more complete representations of human genetic sequence and variation. GRCh38 and recent efforts by the Telomere-to-Telomere (T2T) consortium have clearly shown the limitations of a single reference genome (84). The current T2T assembly, while more comprehensive than GRCh38, also misses genomic segments of diverse human genomes. Pangenome and graph representation efforts (85–87) will provide an interesting framework, with major challenges for the mapping and representation of existing gene and transcript annotations, sequence conservation, or biochemical readouts (e.g. ENCODE ChIP and DNase data). With only initial mappings of coordinates between pangenomes and GRCh38 being developed, it will be a major effort to develop unbiased CADD scores in such settings.

## Supplementary Material

gkad989_Supplemental_File

## Data Availability

The training and validation data underlying this article are available at https://cadd.bihealth.org/training. Pre-scored whole genome SNV files are available at https://cadd.bihealth.org/download.
