# Supplementary material for: CADD v1.7: using protein language models, regulatory CNNs and other nucleotide-level scores to improve genome-wide variant predictions
Source: Nucleic Acids Res. 2024 Jan 5;52(D1):D1143–54. doi: 10.1093/nar/gkad989 (PMC10767851; doi:10.1093/nar/gkad989)
Supplement: gkad989_Supplemental_File [file gkad989_supplemental_file.pdf]

# **Supplementary Information:** CADD v1.7: Using protein language models, regulatory CNNs and other nucleotide-level scores to improve genome-wide variant predictions

Max Schubach[#], Thorben Maass[#], Lusine Nazaretyan[#], Sebastian Röner and Martin Kircher*

[#] These authors contributed equally

*Corresponding author: martin.kircher@uni-luebeck.de

## Table of Contents

Supplementary Figures ........ 3
Supplementary Figure S1 ........ 3
Supplementary Figure S2 ........ 4
Supplementary Figure S3 ........ 6
Supplementary Figure S4 ........ 7
Supplementary Figure S5 ........ 8
Supplementary Figure S6 ........ 9
Supplementary Figure S7 ........ 10
Supplementary Figure S8 ........ 11
Supplementary Figure S9 ........ 12
Supplementary Figure S10 ........ 13
Supplementary Figure S11 ........ 14
Supplementary Figure S12 ........ 16
Supplementary Tables ........ 17
Supplementary Table S1 ........ 17
Supplementary Table S2 ........ 18
Supplementary Table S3 ........ 19
Supplementary Table S4 ........ 20
Supplementary Note S1: Preparation of test data sets ........ 21
DMS data sets ........ 21
Inframe InDel data sets ........ 21
ClinVar derived data sets ........ 21
ClinVar **+ coding SNV missense ........ 21
ClinVar **+ coding SNV stop gain data set ........ 21
ClinVar **+ coding InDel frameshift data set ........ 21

ClinVar coding frameshift and stop gains data set ........ 22
ClinVar 3' UTR data set for APARENT2 tests ........ 22
ClinVar UTR, intergenic and noncoding transcript for gwRVIS tests ........ 22
ExAC variants with minor allele frequency greater equal 5% (MAF5) data set ........ 22
Allele frequency data set for Zoonomia and Roulette tests ........ 22
Saturation mutagenesis MPRA ........ 22
High confidence variant sets (satmut and satmut_snv) ........ 23
Significant variant set (satmut_significant and satmut_significant_snv) ........ 23
Availability of data sets ........ 23
Supplementary Note S2: Calculating ESM-1v-derived scores from nucleotide changes in VCF ........ 23
Missense score ........ 23
Inframe InDel score ........ 23
Frameshift and stop gain score ........ 24
Supplementary Note S3: Regulatory Sequence Model ........ 24
Training/testing/validation ........ 24
Model type and structure ........ 25
Model optimization ........ 25
Prediction of variants ........ 25
Independent test sets ........ 26
CADD integration ........ 26
CADD evaluation ........ 26
Supplementary Note S4: 3'UTR, noncoding constraint, mutational scores and conservation scores ........ 27
APARENT2 ........ 27
Roulette ........ 27
gwRVIS ........ 27
Zoonomia ........ 27
Supplementary Note S5: Training new CADD models ........ 27
Supplementary Note S6: Performance metrics ........ 28
Spearman and Pearson correlations ........ 28
ROC curves and AUROC values ........ 28
Precision recall curves and average precision scores (APSs) ........ 28
References ........ 30

# Supplementary Figures

## Supplementary Figure S1

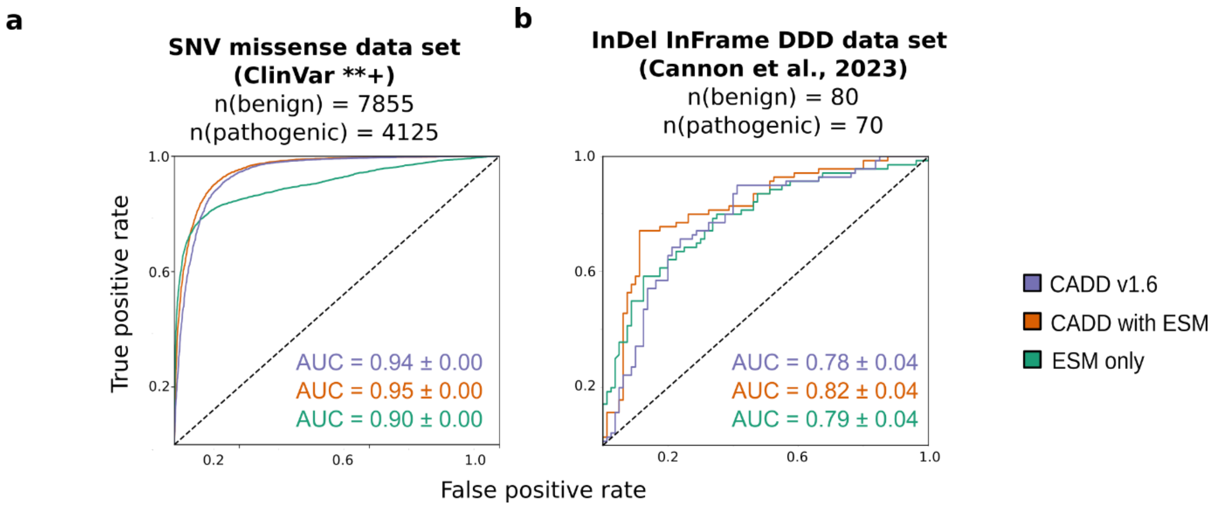


**Figure S1: Performance of CADD employing protein language model derived scores for SNV missense and InDel inframe variants.** (a,b) ROC curves and corresponding AUROC values of CADD v1.6, a CADD model including ESM protein language model score for SNV missense variants (a) or inframe InDel variants (b), and the respective stand-alone ESM protein language model score. The pathogenic class is used as positive class.

# Supplementary Figure S2

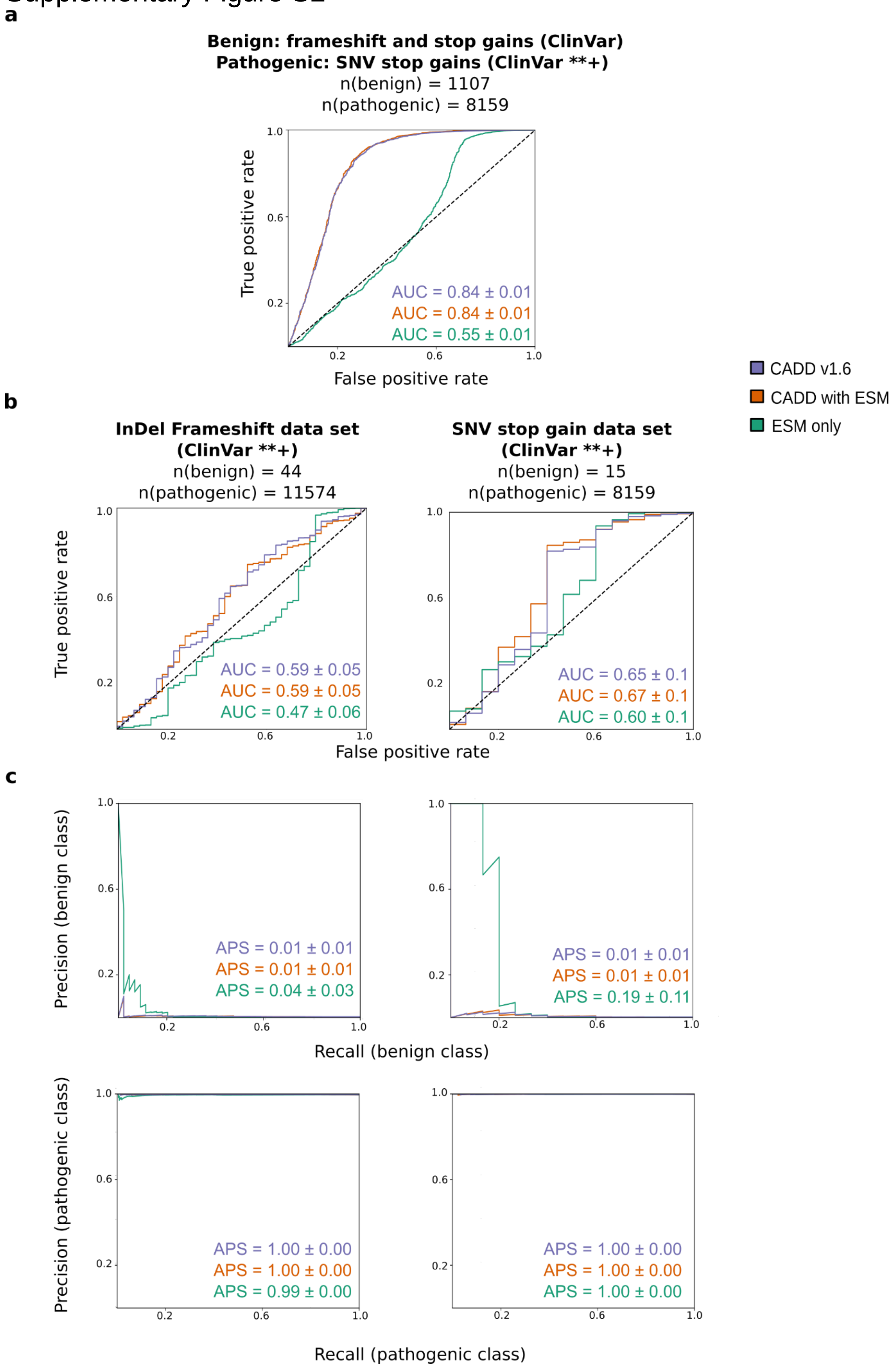

**Figure S2: Performance of CADD employing protein language model derived scores for frameshift and stop gain variants on ClinVar two star plus variants.** (a), (b), and (c) show ROC or precision recall curves and corresponding AUROC or average precision score (APS) values of CADD v1.6, a CADD model including ESM protein language model score for stop gain or frameshift variants and the stand-alone ESM protein language model score. The positive class corresponds to the pathogenic class if not stated otherwise.

Supplementary Figure S3

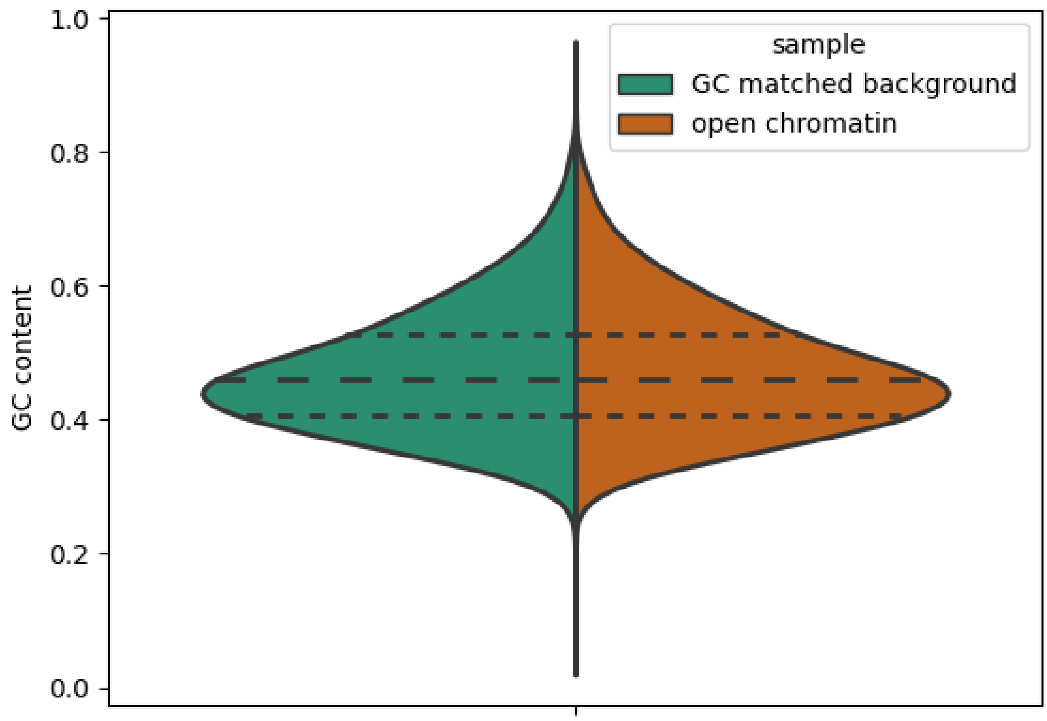


**Figure S3: Distribution of GC content for RegSeq model training data.** Comparison of GC content distribution of the open chromatin data for all seven cell lines compared to random selection of matched GC background sequences. Dashed lines are the quartiles (short dashes) and the median (long dashes). The plot shows the training, test and validation data for 500 bp sequences.

Supplementary Figure S4

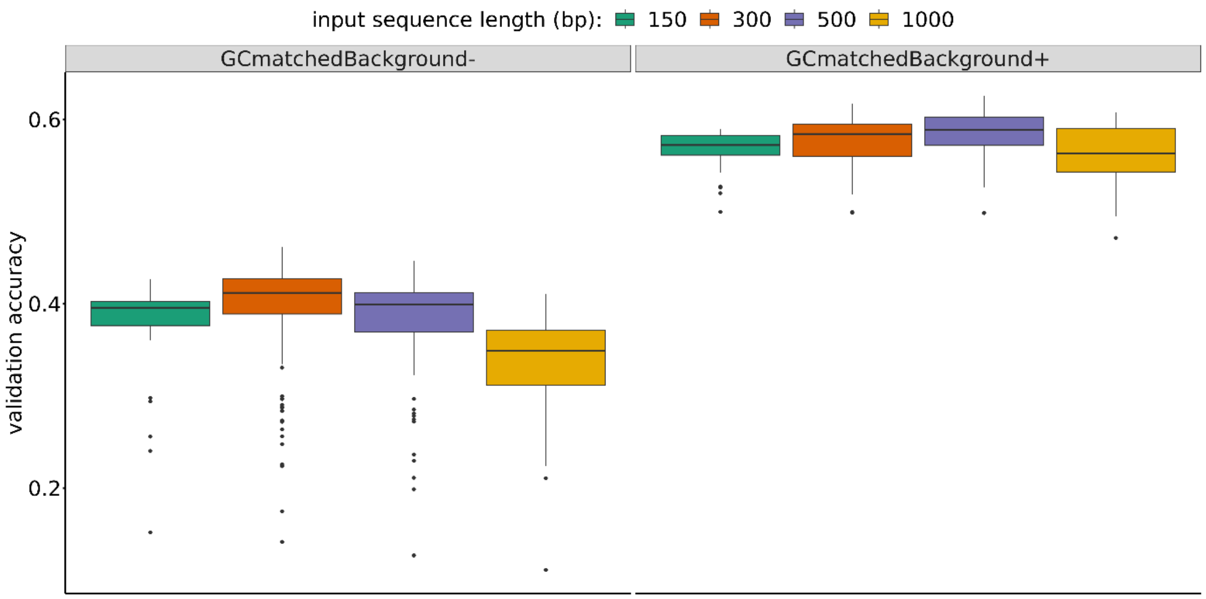


**Figure S4: Validation accuracy of hyperparameter optimization.** Comparing validation accuracy of regulatory sequence multi-task model with GC-matched (GCmatchedBackground+) and without (GCmatchedBackground-) during hyperparameter optimization. Each model went to 400 times of optimization rounds. Validation accuracy is decomposed by sequence lengths in base pairs (bp) chosen by the Tree of Parzen Estimators during optimization rounds.

Supplementary Figure S5

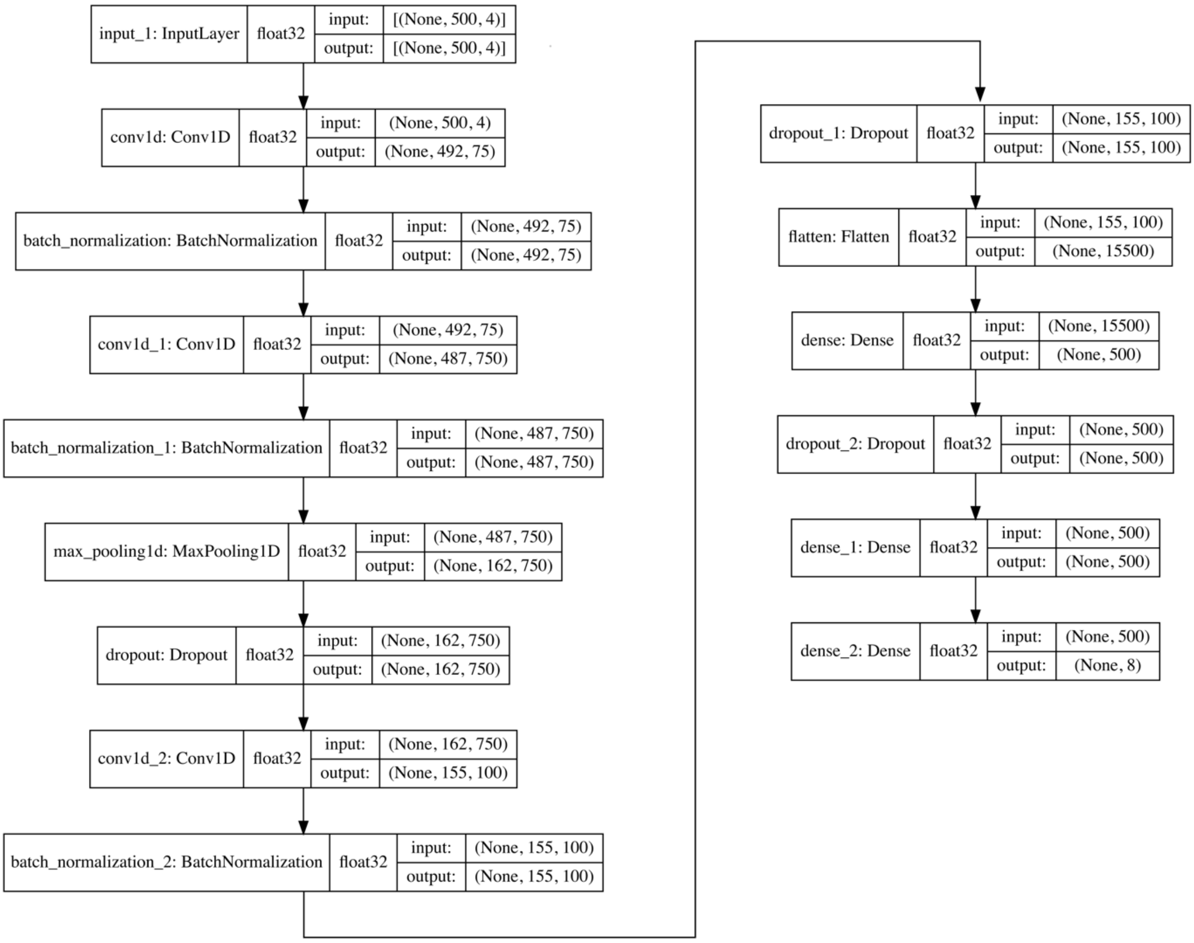


**Figure S5: Schematic overview of RegSeq model.** Visualization of the RegSeq model build in tensorflow using the function *tf.keras.utils.plot_model*.

Supplementary Figure S6

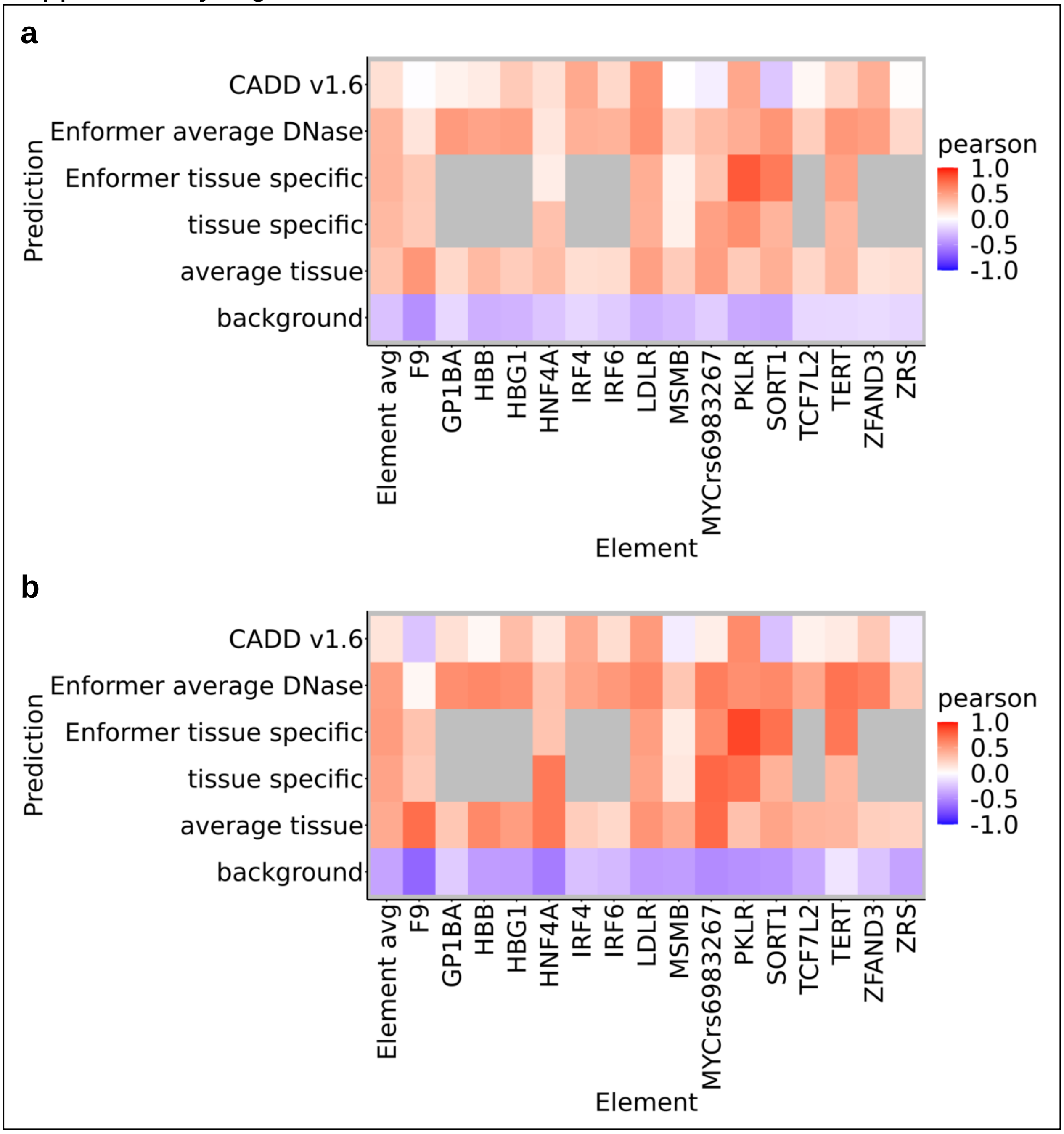


**Figure S6: Pearson correlation to saturation mutagenesis elements.** Correlation of prediction outputs of the regulatory sequence model (RegSeq) by using the alternative minus the reference prediction of the variant. For "tissue specific" prediction only the cell-line output was used where the original experiment was performed. "Average tissue" is the average variant effect over all seven trained cell lines. "Background" is the prediction of the GC-matched negative output. In addition, we show CADD v1.6 (correlated to absolute values of saturation mutagenesis) and Enformer prediction of average DNase tracks ("Enformer average DNase") as well as average tissue specific tracks ("Enformer tissue specific"). "Element avg" is the average correlation for all defined elements of that prediction. Panel **(a)** shows the satmut_snv dataset (all single nucleotide variants with minimum of 10 supported barcodes) and panel **(b)** the satmut_significant_snv dataset (all single nucleotide variants with minimum 10 supported barcodes and p-value $< 10^{-5}$).

Supplementary Figure S7

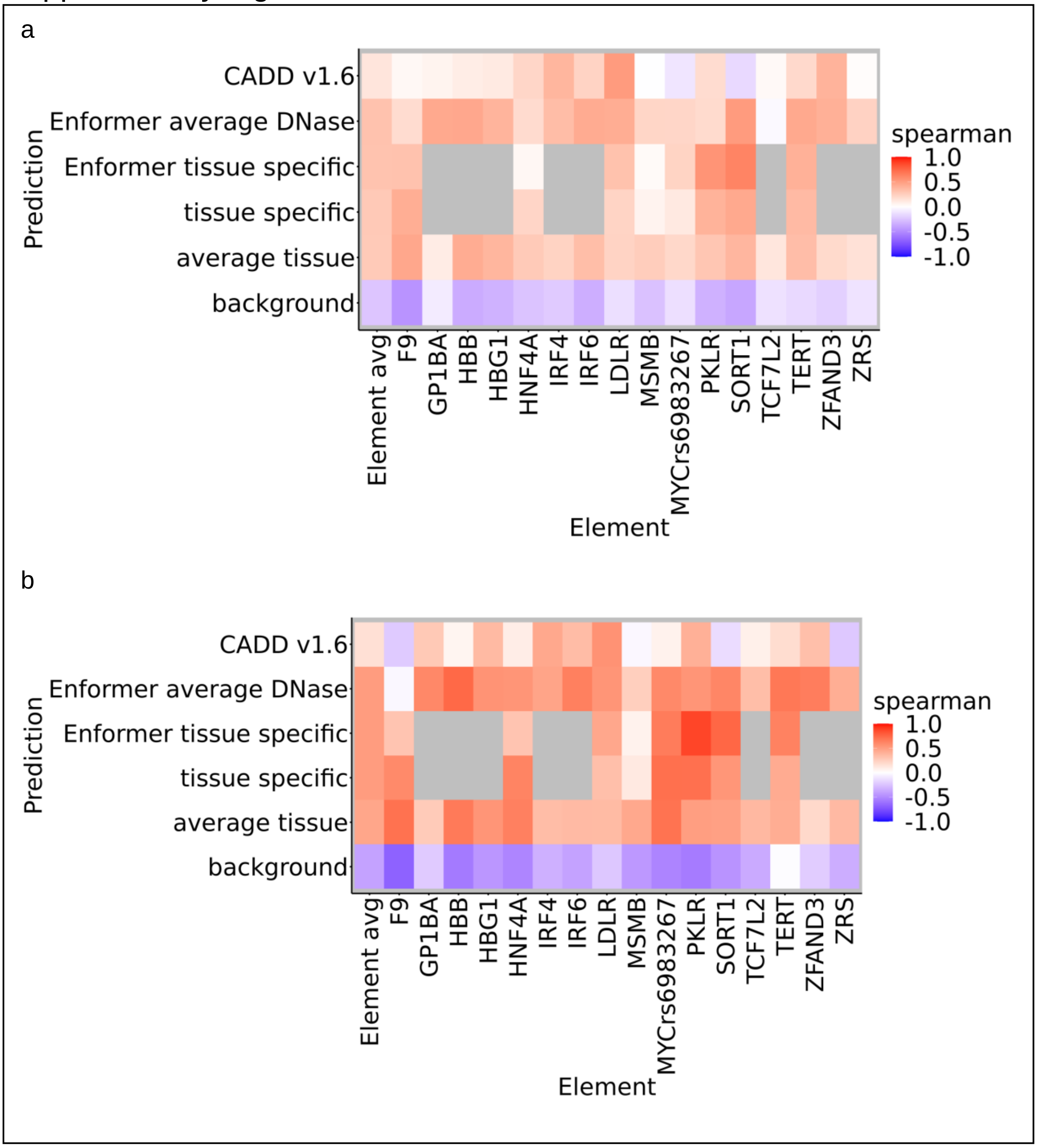


**Figure S7: Spearman correlation to saturation mutagenesis elements.** Correlation of prediction outputs of regulatory sequence model by using the alternative minus the reference prediction of the variant. For "tissue specific" prediction only the cell-line output was used where the original experiment was performed. "Average tissue" is the average variant effect over all seven trained cell lines. "Background" is the prediction of the GC-matched negative output. In addition, we show CADD v1.6 (correlated to absolute values of saturation mutagenesis) and Enformer prediction of average DNase tracks ("Enformer average DNase") as well as average tissue specific tracks ("Enformer tissue specific"). "Element avg" is the average correlation for all defined elements of that prediction. Panel **(a)** shows the satmut_snv dataset (all single nucleotide variants with minimum of 10 supported barcodes) and panel **(b)** the satmut_significant_snv dataset (all single nucleotide variants with minimum 10 supported barcodes and p-value < $10^{-5}$).

# Supplementary Figure S8

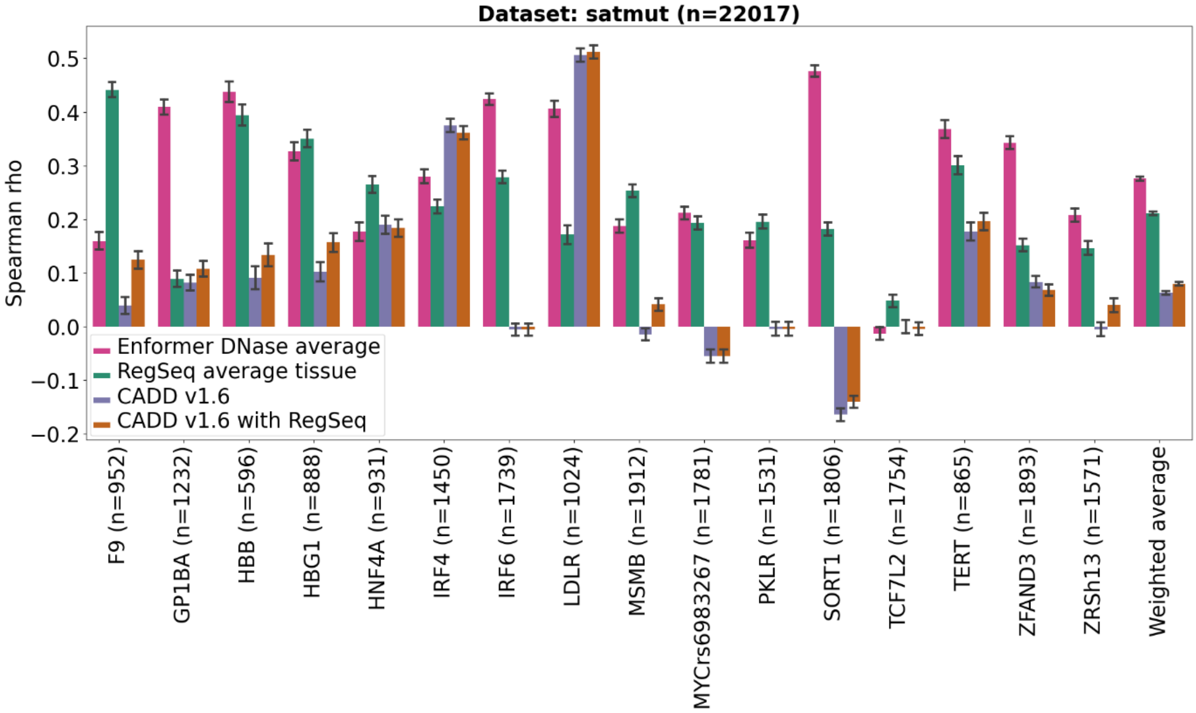


**Figure S8: Spearman correlation to saturation mutagenesis elements after bootstrapping.** Correlation of prediction outputs of CADD v1.6 with integrated RegSeq features in comparison with CADD v1.6, the original RegSeq features (average of RegSeq{0..6} and RegSeq "average tissue") and Enformer average DNAse prediction. For both CADD-based scores we used the absolute value of the MPRA effect because CADD is not expected to predict effect directionality. Weighted average is the weighted mean of Spearman correlation by the number of variants (denoted by n) across all elements. Dataset satmut (1 bp deletions and SNVs) with a minimum support of 10 barcodes is shown. Bars show the average Spearman correlation after bootstrapping (1000 runs with 80% of the element variants). Error bars define the standard deviation across runs.

## Supplementary Figure S9

a

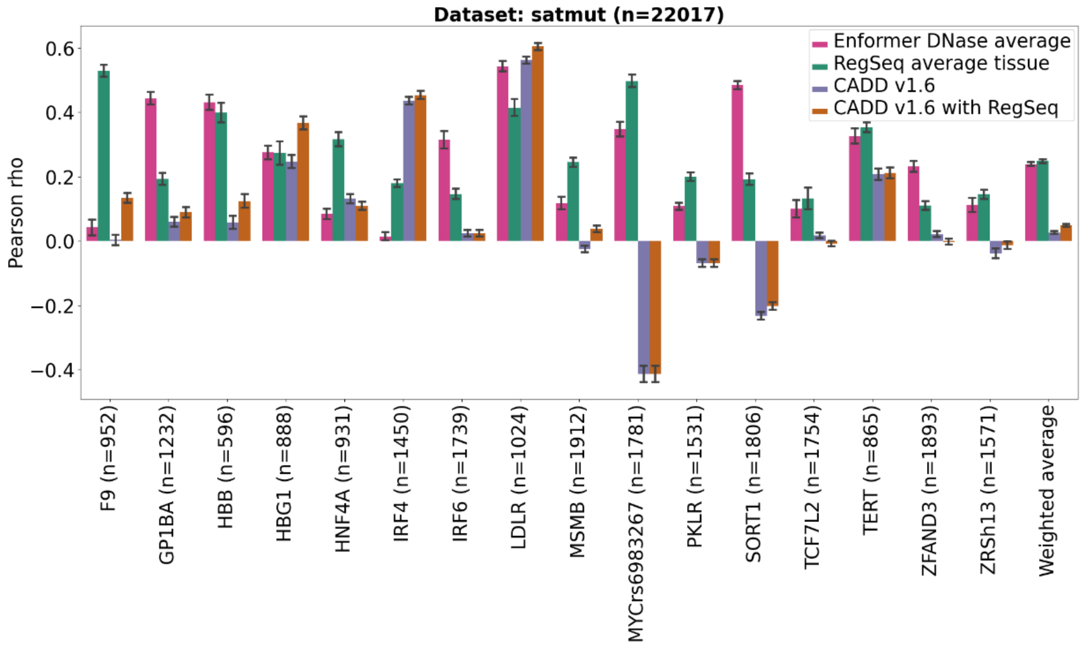


b

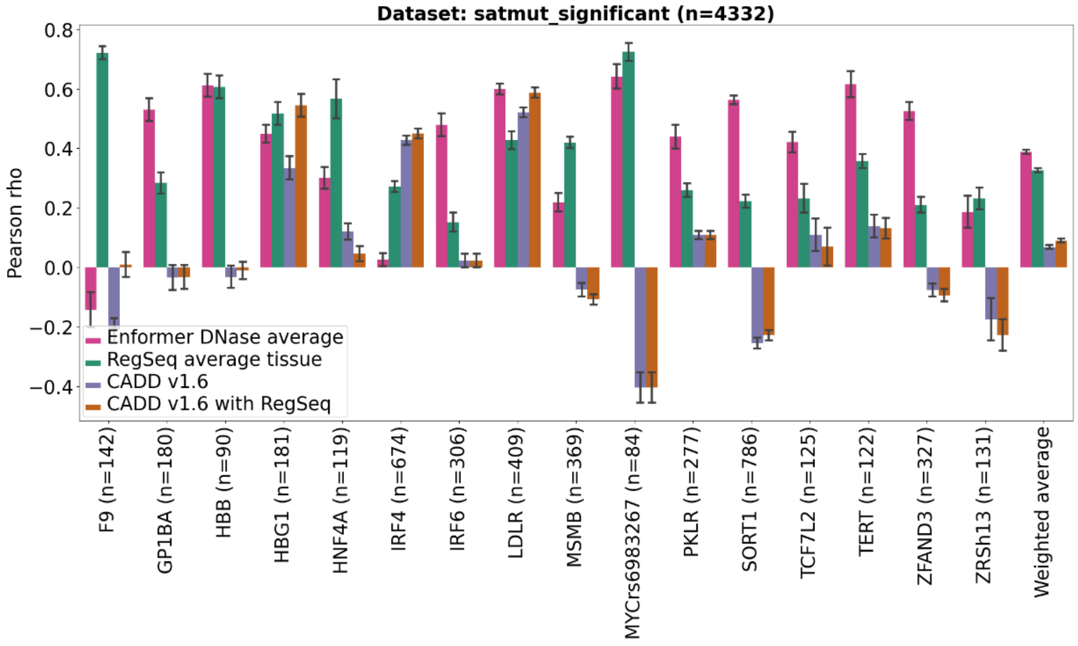


**Figure S9: Pearson correlation with saturation mutagenesis MPRA data.** Correlation of prediction outputs of CADD v1.6 with RegSeq features in comparison with CADD v1.6, the original RegSeq features (average of RegSeq{0..6} and RegSeq "average tissue") and Enformer average DNAse prediction. For CADD v1.6 with RegSeq and CADD v1.6, we used the absolute value of the MPRA effect as CADD is not expected to predict the directionality of the effect. Weighted average is the weighted mean of the Pearson correlation by the number of variants (denoted by n) across all elements. Panel **(a)** visualizes the dataset satmut (1 bp deletions and SNVs with minimum of 10 barcodes). Panel **(b)** visualizes the satmut_significant dataset (1 bp deletions and SNVs with minimum 10 barcodes and p-value smaller < $10^{-5}$). Bars show the average Pearson correlation after bootstrapping (1000 runs with 80% of the element variants). Error bars define the standard deviation across runs.

# Supplementary Figure S10

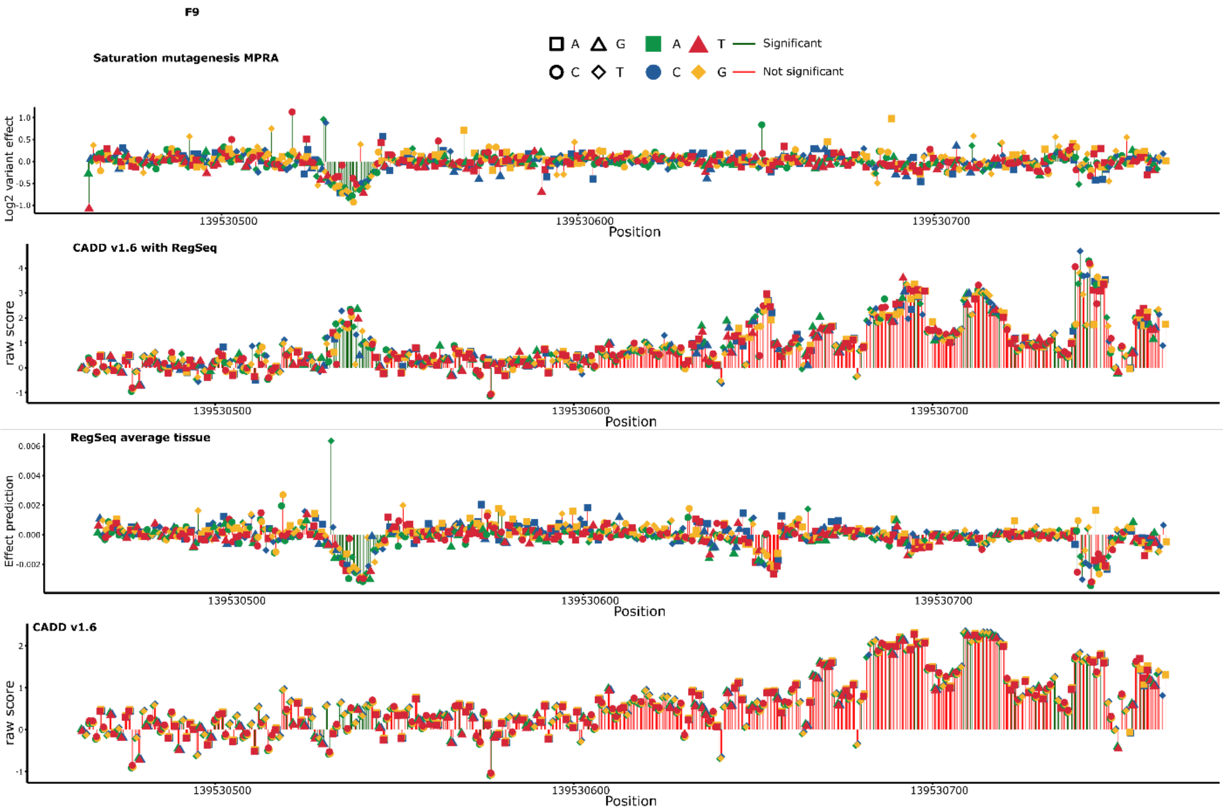


**Figure S10: Predictions variant effects at the *Factor IX (F9)* promoter.** Comparison of the original variant effects to CADD with RegSeq features, the RegSeq model predictions of variant effects and the original CADD v1.6.

## Supplementary Figure S11

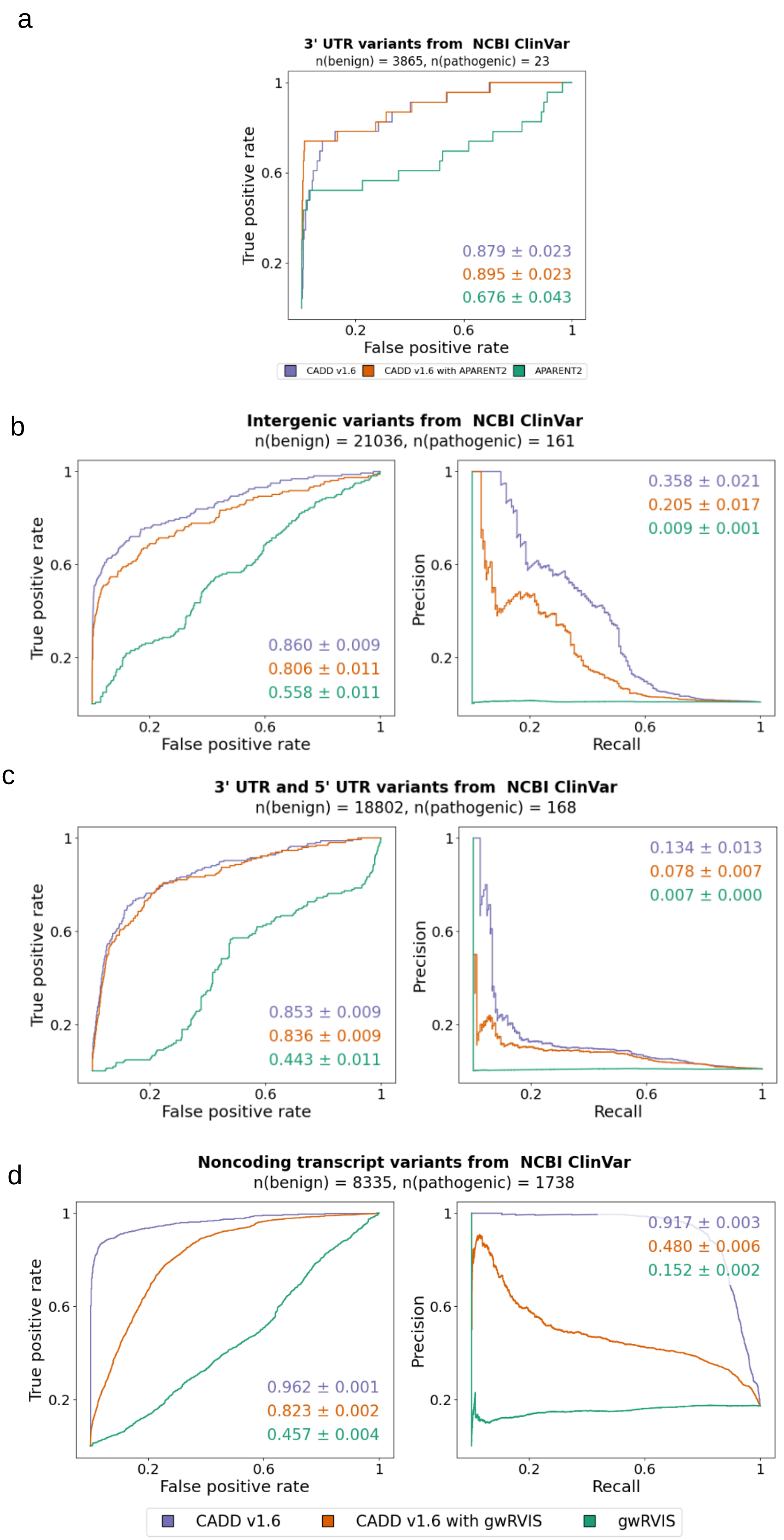


**Figure S11: Comparison of CADD performance with and without APARENT2 and gwRVIS**. **(a)** Recall curves of CADD v1.6, a CADD model with APARENT2 and only APARENT2 scores on 3'UTR variants from the NCBI ClinVar dataset. CADD recall increases with the integration of the APARENT2 score. **(b)-(d)** Recall and precision-recall curves on the NCBI ClinVar dataset for CADD v1.6, CADD with gwRVIS as well as only gwRVIS-scores for intergenic, 3' und 5'UTR and noncoding transcript variants. For all three variant groups, the model performance deteriorates with integration of gwRVIS.

# Supplementary Figure S12

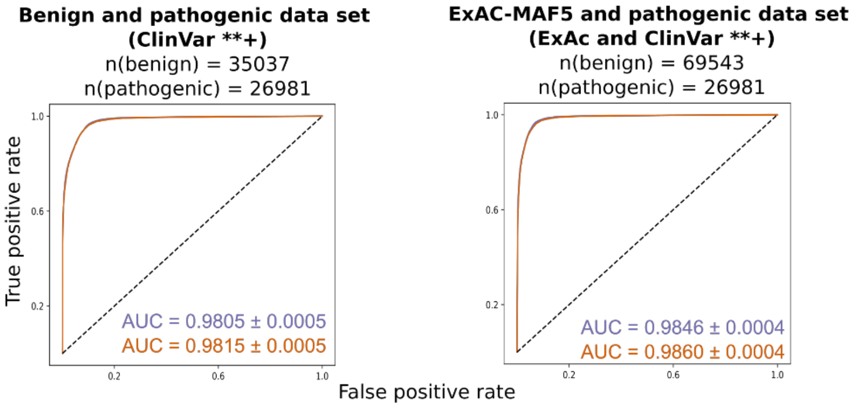


**Figure S12: Evaluation of CADD v1.7 performance on NCBI ClinVar derived variant sets.** Performance of CADD v1.6 (purple) and v1.7 (orange) on separating pathogenic variants from ClinVar with a two star plus reviewer status from ClinVar benign *(left)* or variants of the ExAC MAF5 data set *(right)*.

# Supplementary Tables

## Supplementary Table S1

Scores and annotations tested for integration in CADD v1.7. The last column indicates whether the annotation was included in CADD v1.7. A full list of annotations already used for CADD v1.6 can be found in Supplementary Tables S1 and S2 in Rentzsch et al., 2021 (1).

| Model | Description | Genome region | Annotation | Reference | CADD v1.7 |
|---|---|---|---|---|---|
| ESM-1v | Meta AI Evolutionary Scale Model for variant effects in protein coding sequences | coding | missense<br>InDels Inframe<br>InDels Frameshift<br>stop gain | (2) | Yes |
| RegSeq | CNN trained on open chromatin sequences of multiple tissues | genome-wide | RegSeq0 - HEK293T<br>RegSeq1 - K562<br>RegSeq2 - HepG2<br>RegSeq3 - HeLa-S3<br>RegSeq4 - MC-7<br>RegSeq5 - iPS DF 19.11<br>RegSeg6 - GM23338<br>RegSeg7 - GC-matched background | In-house development | Yes |
| APARENT2 | human polyadenylation | 5' and 3'UTR | APARENT2 score | (3) | Yes |
| Zoonomia | Conservations scores | genome-wide | Primate PhyloP (43 genomes) scores<br>Zoonomia PhyloP (241 genome) scores<br>UltraConserved Elements (UCE)<br>Runs of Contiguous Constraint (RoCC) | (4) | Yes |
| Roulette | Mutability score | genome-wide | MR: Roulette mutation rate estimate<br>AR: Adjusted Roulette mutation rate estimate<br>Filter:<br>• low - Low quality regions by gnomAD<br>• high - High quality regions by gnomAD<br>• TBFS - Transcription factor binding site as determined by overlap with ChIP-seq peaks.<br>• SFS_bump - Pentamer context with abnormal site frequency spectra (SFS) | (5) | Yes |
| gwRVIS | variation intolerance score for non-coding regions | non-coding | gwRVIS-score<br>gwRVIS-probability | (6) | No |

## Supplementary Table S2

ENCODE IDs of narrow peak files as well as the ID of the corresponding DNase-seq experiment used as initial DNase-seq regions for training. Regions are on human genome reference build GRCh38.

| Cell-Line | ENCODE ID: experiment | ENCODE ID: narrow peak file |
|---|---|---|
| HEK293T | ENCSR000EJR | ENCFF127KSH |
| K562 | ENCSR921NMD | ENCFF248FIZ |
| HepG2 | ENCSR000ENP | ENCFF470WDP |
| | | ENCFF630RQY |
| | ENCSR149XIL | ENCFF209DJG |
| | | ENCFF711IED |
| HeLa-S3 | ENCSR959ZXU | ENCFF950NDW |
| | | ENCFF736UEX |
| MCF-7 | ENCSR000EPJ | ENCFF751SAV |
| | | ENCFF636FXP |
| | ENCSR000EPJ | ENCFF961ZCT |
| | | ENCFF229SJW |
| iPS DF 19.11 | ENCSR383SNM | ENCFF607FJQ |
| GM23338 | ENCSR004SUL | ENCFF525NNH |
| | | ENCFF666TNT |

## Supplementary Table S3

Results of the hyperparameter optimization after 400 training rounds using Tree of Parzen Estimators (7) by the software hyperopt (8).

| Layers: | Input | Convolution 1; Convolution 2 | | | Max Pooling 1 | | Dropout 1 | Convolution 2; Convolution 3 | | | Max Pooling 2 | | Dropout 2 | Dense 1 | | Dropout 3 | Dense 2 | |
|---|---|---|---|---|---|---|---|---|---|---|---|---|---|---|---|---|---|---|
| Model | | Filter | Kernel size | activation | Pool size | stride | | Filter | Kernel size | activation | Pool size | stride | | output | activation | | output | activation |
| Multitask without negatives | 300 | 750; 500 | 8;8 | relu; relu | - | - | 0.3 | 250; 50 | 4;3 | relu; relu | 2 | 2 | 0.3 | 500 | Sigmoid | - | 300 | sigmoid |
| Multitask with negatives | 500 | 75; 750 | 9;6 | relu; softmax | 3 | None | 0.3 | 100;- | 4;- | softmax | - | - | 0.3 | 500 | sigmoid | 0.2 | 500 | sigmoid |

## Supplementary Table S4

New CADD features derived from the regulatory sequence model.

| Feature name | Transformation | Default | Description |
|---|---|---|---|
| regseq_positivemax | max([x if x>0 else 0 for x in Regseq0,..,RegSeq6 ]) | 0 | Maximum positive value of cell-type variant effects |
| regseq_negativemax | abs(max([x if x<0 else 0 for x in RegSeq0,..,RegSeq6 ])) | 0 | Absolut maximum negative value of cell-type variant effects |
| regseq_positivemin | min([x if x>0 else 0 for x in RegSeq0,..,RegSeq6 ]) | 1 | Minimum positive value of cell-type variant effects |
| regseq_negativemin | abs(min([x if x<0 else 0 for x in RegSeq0,..,RegSeq6 ])) | 1 | Absolut minimum negative value of cell-type variant effects |
| regseq_positivemean | mean([x if x>0 else 0 for x in RegSeq0,..,RegSeq6 ]) | 0 | Average of positive of cell-type variant effects |
| regseq_negativemean | abs(mean([x if x<0 else 0 for x in RegSeq0,..,RegSeq6 ])) | 0 | Absolute average of positive cell-type variant effects |
| regseq_positivestd | stdev([x if x>0 else 0 for x in RegSeq0,..,RegSeq6 ]) | 0 | Standard deviation of positive cell-type variant effects |
| regseq_negativestd | stdev([x if x<0 else 0 for x in RegSeq0,..,RegSeq6 ]) | 0 | Standard deviation of negative cell-type variant effects |
| regseq_positive | RegSeq7 if RegSeq7>0 else 0 | 0 | Positive GC matched background prediction |
| regseq_negative | abs(RegSeq7 if RegSeq7<0 else 0) | 0 | Negative GC matched background prediction |

# Supplementary Notes

## Supplementary Note S1: Preparation of test data sets

### DMS data sets

DMS scores, UniProt IDs, and the corresponding amino acid substitutions were taken from the ProteinGym database (9). As CADD works on genomic coordinates and requires a file in Variant Call Format (VCF) as input, amino acid substitutions from the ProteinGym were translated to nucleotide substitutions. For this purpose, the UniProt ID mapping tool (10) was used to translate UniProt IDs to Ensembl Gene IDs. We then used the GRCh38 CADD v1.6 genome-wide SNVs CADD score file with annotations (including nucleotide substitutions, chromosome position, the Ensembl VEP tool's amnio acid substitution and Gene ID annotations) and filtered for the respective Ensembl Gene ID, missense consequence and matching amnio acid substitution given in the ProteinGym database. We linked DMS scores with information on chromosome position and nucleotide substitution. We only considered amino acid substitutions that are possible due to an SNV. As different SNVs can result in the same amino acid substitutions, we used substitutions corresponding to the highest CADD v1.6 score.

### Inframe InDel data sets

We used a previously curated set of inframe insertion and deletion changes (11). The entire dataset was downloaded from https://doi.org/10.1186/s12920-023-01454-6. The data set was split into benign and likely benign variants and pathogenic and likely pathogenic variants. Chromosome positions based on the GRCh38 genome build, reference and alternative alleles were readily obtained from the data set's annotations. The Deciphering Developmental Disorders (DDD) subset was obtained by using the respective annotation in the data set.

### ClinVar derived data sets

We used the NCBI ClinVar database (12) in its June 2023 release (https://ftp.ncbi.nlm.nih.gov/pub/clinvar/tab_delimited/archive/variant_summary_2023-06.txt.gz) as the basis of multiple validation sets and filtered for pathogenic, likely pathogenic, likely benign, and benign SNVs and InDels < 50 bp. To generate input files for our CADD workflow, we used the fields chromosome, chromosome position, reference allele, and alternative allele according to the GRCh38 genome build.

#### *ClinVar **+ coding SNV missense*

The basic data set was further filtered for variants annotated with either benign or pathogenic with a two star and higher reviewer status ("**+") and split into InDel variants and SNVs. SNVs were annotated using the CADD v1.6 webserver employing the GRCh38 genome build and subsequently filtering for variants annotated as missense.

#### *ClinVar **+ coding SNV stop gain data set*

Like for the missense variants above, a set filtered for stop gain consequence was obtained.

#### *ClinVar **+ coding InDel frameshift data set*

The InDel variants in the basic data set with a two star plus reviewer status ("**+") were annotated using the CADD v1.6 webserver (GRCh38 genome build) and subsequently filtered for a frameshift consequence.

*ClinVar coding frameshift and stop gains data set*
Benign variants from the basic data set were annotated using the CADD v1.6 webserver (GRCh38 genome build) and subsequently filtered for variants annotated with either stop gain or frameshift consequence.

*ClinVar 3' UTR data set for APARENT2 tests*
All variants in the basic ClinVar dataset described above were scored using CADD v1.6 (GRCh38 genome build) and subsequently filtered for variants annotated with a 3'UTR consequence and for that an APARENT2 score was available.

*ClinVar UTR, intergenic and noncoding transcript for gwRVIS tests*
The basic ClinVar dataset was annotated using CADD v1.6 (GRCh38) and filtered for variants using either the 'Consequence' annotations of 3' or 5'UTR or by using the 'AnnoType' annotations Intergenic or Noncoding. In addition, only variants for that a gwRVIS score was available were considered.

*ClinVar **+ benign and pathogenic data set*
The basic data set was further filtered for variants annotated with either benign or pathogenic with a two star and higher reviewer status ("**+").

## ExAC variants with minor allele frequency greater equal 5% (MAF5) data set

Proxy-benign variants from efforts of the Exome Aggregation Consortium with a minor allele frequency of at least 5% were obtained from our previously released validation sets (13). Specifically, the data set was downloaded from https://krishna.gs.washington.edu/download/CADD-development/v1.4/validation/clinVar-ExAC/. The data set was lifted to GRCh38 using CrossMap and the Ensembl Assembly converter tool (https://www.ensembl.org/Homo_sapiens/Tools/AssemblyConverter). Variants that also occur in the ClinVar **+ pathogenic data set (see above) were removed.

## Allele frequency data set for Zoonomia and Roulette tests

100,000 SNVs and 100,000 InDels were sampled from the variant release 3 (file: 20110521) dataset of the 1000 Genomes Project (14). The minor allele frequencies of those variants were correlated with the respective CADD scores.

## Saturation mutagenesis MPRA

For evaluation of regulatory variant effects, we use massive parallel reporter assay (MPRA) data of twenty saturation mutagenesis promoter or enhancer elements (15). We only use regulatory elements with high correlation across replicates of the MPRA experiments (dropping MYCrs11986220, BCL11A, FOXE1 and UC88). When multiple experiments of the same element were performed in the study, we used the experiment with the highest replicate correlation. However, for TERT the experiment in HEK293T was used as the HEK293T cell-type is also included in our RegSeq model (see Supplementary Note S3). This left us with 16 elements that are used for a cell-type agnostic comparison (F9, GP1BA, HBB, HBG1, HNF4A, IRF4, IRF6, LDLR.2 called LDLR, MSMB, MYCrs6983267, PKLR 48h called PKLR, SORT1, TCF7L2, TERT-HEK called TERT, ZFAND3, ZRSh13). Promoter sequence experiments that matched our cell lines are F9, HNF4A, LDLR, MSMB, PKLR, and TERT-HEK and the enhancers experiments that matched our cell lines are MYCrs6983267 and SORT1. Those were inspected separately matching the cell line prediction. To use only robust variant effects, we further filtered the variants of the saturation mutagenesis data as described below.

*High confidence variant sets (satmut and satmut_snv)*
Here we select only variants with a minimum of 10 MPRA barcodes (dataset satmut, 22,017 variants across all elements). For some comparison we selected only SNVs and removed all 1 bp deletions (dataset satmut_snv, 20,492 SNVs).

*Significant variant set (satmut_significant and satmut_significant_snv)*
In addition to the barcode threshold, we looked at the subset of significant (non-zero) variant effects with a p-value of lower than $10^{-5}$ (dataset satmut_significant and satmut_significant_snv). This results in 4,332 number of variants across all elements (4,141 SNVs).

## Availability of data sets

Variants in the above-described test data sets are available as VCF files on our webserver (https://kircherlab.bihealth.org/download/CADD-development/v1.7/validation/).

# Supplementary Note S2: Calculating ESM-1v-derived scores from nucleotide changes in VCF

ESM-1v protein language models (2) require an amino acid sequence as sole input and return log-transformed probabilities for each possible amino acid at a given position based on the entire remaining primary sequence. To incorporate ESM-1v-derived scores into CADD, a particular variant in VCF format (i.e., nucleotide level) was first annotated with its Ensembl Transcript ID, a consequence and an amino acid substitution using the Ensembl VEP annotations (16), which are part of regular CADD scoring.

## Missense score

Scores for missense variants were calculated for variants annotated with Ensembl VEP missense consequence. Stop gain, stop lost, and stop retained consequences were explicitly excluded. Multiple amino acid substitutions were treated as inframe InDel (see below). The amino acid sequence was obtained by matching the Ensembl Transcript ID with entries in a FASTA file containing amino acid sequences of all Ensembl transcripts (http://ftp.ensembl.org/pub/release-110/fasta/homo_sapiens/pep/Homo_sapiens.GRCh38.pep.all.fa.gz). Additional stop codons present in the sequence were removed and the amino acid substitution centered within a 350 amino acid window. As final score, we used the averaged log odds ratio between the alternative and reference amino acid employing the five different ESM-1v models: esm1v_t33_650M_UR90S_1, esm1v_t33_650M_UR90S_2, esm1v_t33_650M_UR90S_3, esm1v_t33_650M_UR90S_4, and esm1v_t33_650M_UR90S_5.

## Inframe InDel score

Scores for inframe InDel variants were calculated for variants annotated with the Ensembl VEP consequence inframe insertion or deletion, or if multiple amino acids were substituted. Variants with stop gain, stop lost, and stop retained consequence annotations were explicitly excluded. Amino acid sequences of reference alleles were obtained as described above, with the only difference that a window of 250 amino acids was used. As for InDel variants multiple amino acids can be affected, the amino acid sequence corresponding to the alternative allele can differ in more than one position from the sequence of the reference allele. To account for this, we obtained the entire amino acid sequence of the alternative and reference sequence from Ensembl VEP. To calculate scores for inframe InDel variants, log-transformed probabilities of the entire reference and alternative sequences were added up, respectively,

and subtracted from each other, yielding log odds ratios. The log odds ratios resulting from each of the five models (see above) were than averaged and used as final score.

## Frameshift and stop gain score

Scores for frameshift or stop gain variants were calculated for variants annotated with the Ensembl VEP frameshift or stop gain consequence. Amino acid sequences of reference alleles were obtained as described above, with the only difference that a window of 250 amino acids was used. Log-transformed probabilities were calculated and summed up for the entire reference amino acid sequence. Calculation of a score for the alternative allele was carried out based on the entire reference amino acid sequence. Here, we summed up log probabilities of the reference sequence's amino acids up to the point where the frameshift or stop gain occurred as obtained from Ensembl VEP annotations. For every amino acid that is lost due to the frameshift or stop gain, we used the median of log transformed probabilities calculated from all possible amino acids at each individual position in the remaining sequence and added them to the sum corresponding to the alternative allele. The average of logs odds ratios between the reference and alternative sequences from the five models (see above) was then used as a final score.

# Supplementary Note S3: Regulatory Sequence Model

The model was trained and optimized on open chromatin data from seven ENCODE cell lines. DNase-seq data of five frequently studied cell lines in ENCODE (HEK293T, K562, HepG2, HeLa-S3, and MCF-7) and two induced pluripotent stem cells (iPS DF 19.11 and GM23338) are used for model training (see Supplementary Table S2). Narrow peaks of the DNase hotspot pipeline of ENCODE were downloaded (GRCh38) and the summit of the peaks selected. If multiple runs/experiments/replicates per cell-line were available, peaks overlapping 100 bp left/right the peak summit are merged using BEDtools (17) (i.e., bedtools merge -d 100) and the new center is used. Experiments of a cell-line with insufficient read length defined by ENCODE (min. 36 bp), which leads to lower spot-scores, were excluded. Only regions on autosomes and gonosomes, excluding alternative contigs, were used.

Center/summit regions of all cell lines are combined and centers/summits with an overlap after extension by 50 bp merged (bedtools merge -d 50). The new interval center will be used. Afterwards, centers/summits are extended to 150, 300, 500, or 1000 bp (e.g., for 300 bp: bedtools slop -l 150 -r 149). A region will be positive for a cell line when their original interval overlaps at least 80% with the new combined interval (label 1). Otherwise, it will be negative (label 0).

Models were trained with and without additional negative examples. The additional negative region set has the same size as the union of all positives with the exact same GC distribution (visualized for 500 bp in Supplementary Figure S3). For this purpose, the reference genome is partitioned into 150, 300, 500, and 1000 bp windows using a sliding window of 50 bp (e.g., for 300bp, bedtools makewindows -w 300 -s 50). Regions overlapping (1bp or more) with the positive regions are removed (bedtools intersect -v). From this region set a subset of regions are selected with same size and same GC distribution (1:1 match) using the negative_training_sampler v0.3.0 (https://github.com/kircherlab/negative_training_sampler).

## Training/testing/validation

Final training data was augmented by using forward and reverse sequences. Sequences are one-hot encoded, including N as [0,0,0,0]. Regions on chromosomes other than 8 and 18 are used for training. All regions on chromosome 18 are used as validation set and regions on

chromosome 8 as test set. Early stopping on the validation set is used with patience of 10, restoring the best weights to prevent overfitting of the network.

## Model type and structure

We build a multi-task model using all cell lines. The model input layer is a 4x1 tensor of the length of the input sequence (one-hot encoded sequence). The input layer is followed by one or two blocks of two convolutional layers with similar kernel sizes in a block as well as a maxpooling and dropout layer at the end of each block, depending on the hyperparameter search. After flattening the convolutional outputs, two dense layers, inserting a dropout layer in between, follow. The output is retained linear without any transformation function. The output of this multi-task model has the length of the number of tissues used. When training was done with the additional negative region set the corresponding labels are added to the output.

## Model optimization

Structure and parameters of the deep neural net are optimized using Tree of Parzen Estimators (7) by the software hyperopt (8). The input sequence is optimized using sequence lengths of 150, 300, 500, or 1000 bps. For the convolutional layers we optimized the number of layers (1-4), the kernel size (6 to 10 for the first two layers, 2-4 for the second two layers), the activation function (sigmoid, relu, or softmax), and the number of filters (750, 500, 300, 250, 200, 150, 100, 75, or 50). Stride was always set to one. After two and four convolutional layers, we optimized if we include maxpooling (pooling: 1 to 3, stride: no stride, 1, or 2) with or without a dropout of 0.1, 0.2, or 0.3. For the two dense layers, we optimized the activation function (sigmoid, softmax) and the number of outputs (100, 200, 300, 400, or 500). In addition, we studied whether an additional dropout increases the performance after the first dense layer (dropout value 0.1, 0.2, 0.3, 0.4, or 0.5). In total we ran 400 training iterations maximizing the accuracy on the validation set. For each iteration, we used early stopping with a patience of 5 and allowing a minimum accuracy delta of 0.001 to judge the results as equal. The best model architecture after 400 training iterations is shown in Supplementary Table S4. We dissected the runs where hyperopt used different input lengths and inspected the validation accuracy (see Supplementary Figure S4 and Table S3). In general, the multi-task model including negative GC matched background outperforms the other, which we already described in a previous study (18). Interestingly, the multi-task model with additional GC-matched negative regions used favors sequence lengths of 500bp compared to the model without, which favors sequence lengths of 300bp (compare with Supplementary Figure S4). It seems that the surrounding sequence is important to distinguish "positive" and "negative" labels. Longer sequence length (1000bp) did not improve models and was worse than very short fragments. From these results we selected the best performing model (Supplementary Figure S4) with negatives to continue with further analysis. A schematic overview of the model can be found in Supplementary Figure S5.

## Prediction of variants

Variants effects were predicted by *in-silico* mutagenesis. Here, sequences are extended to both sides to fit the model input (e.g., bedtools slop -l 250 -r 249 for a 500bp input in case of a SNV). Then the reference input sequence and the alternative (containing the new allele) input sequences are fed to the model. The variant effect is defined as the prediction output of a task for the alternative minus reference sequence (delta with sign). Given the different output tasks, our model generates eight outputs for each variant (called RegSeq0 to RegSeq7).

## Independent test sets

We use MPRA data to benchmark our model and later the integration into CADD. We use saturation mutagenesis data as described in Supplementary Note S1. We used the enhancer/promoter elements conducted in the cell lines used for training to measure cell line/tissue-specific performance. For a general (average) prediction over all experiments (regardless of the cell line) we selected all elements as defined in Supplementary Note S1. Prediction on these elements was done using the average variant effect prediction of all seven cell lines and in addition the negative channel. Because all saturation mutagenesis experiments were done separately, we correlated each element separately and used an average correlation across elements for average performance.

In Supplementary Figure S6 we show Pearson and in Supplementary Figure S7 Spearman correlation of datasets satmut_snv and satmut_significant_snv of our regulatory sequence model using tissue specific predictions, average predictions over the seven cell lines as well as the GC-matched background prediction. We contrast the scores with the original CADD v1.6 predictions and the Enformer output of average DNase tracks (average DNase predictions of the same cell line for tissue-specific elements). Absolute values of the expression gain and losses should correlate with CADD's deleteriousness predictions. We see that our regulatory sequence model performs like Enformer and has improved predictions compared to CADD v1.6. The negative matched background prediction of the model is negatively correlated and therefore provides additional information. Comparing tissue specific predictions with average predictions over the seven tracks, we do not see an overall benefit for those elements. Performance depends on the element and might be related to quality differences for the different cell lines in the training data. Because CADD is tissue agnostic, we decided to use average predictions across cell lines.

## CADD integration

We use the prediction difference between alternative and reference sequence (variant centered within the 500bp) for the seven cell lines (RegSeq0, RegSeq1, …, RegSeq6) and the GC-matched background as new input features for CADD (RegSeq7). We apply transformations on those values before training/prediction as the directionality of variant effects (e.g., motif gain/loss) does not align with a linear modeling and organismal nature of CADD scoring. For the cell line predicted variant effects (RegSeq{0..6}), we use the maximum, minimum, mean value, and standard deviation of all positive variant effects and separately of all negative variant effects (otherwise 0). This results in eight features per variant, for which absolute values are considered in the logistic regression. The negative background (RegSeq7) is considered as two features (absolute value of positive or negative effect). Default values are zero except for the minimum feature, where we use one as the default. In total this results in ten new CADD training features which are listed in Supplementary Table S4. During training we flipped reference and alternative sequence for human-derived variants to ensure the correct directionality of the sequence change (i.e., ancestral sequence to the human reference allele).

## CADD evaluation

We trained CADD v1.6 with the described RegSeq features and correlated the output with the saturation mutagenesis MPRA readouts. Figure 3 shows the Spearman correlation of confident variants with a significant experimental effect (Supplementary Note S1), before and after integration of RegSeq features. Correlation of all confident variants is available in Supplementary Figure S8 and S9. We see an improved performance over nearly all elements as well as an overall improvement over the regulatory variant effects and conclude that the

new sequence-based regulatory features are incorporated by the logistic regression model and improve CADD's regulatory variant effect prediction.

# Supplementary Note S4: 3’UTR, noncoding constraint, mutational scores and conservation scores

## APARENT2

APARENT2 scores were downloaded from https://drive.google.com/drive/folders/1rg7VHKBM19iFIruzDgQ4BtUVqgcjypxu and filtered for the GRCh38 genome build. If for one position and one gene, multiple scores were available, the maximum value was retrieved.

## Roulette

Roulette scores were downloaded from http://genetics.bwh.harvard.edu/downloads/Vova/Roulette/, converted into a tab delimited files using vcf2tsvpy (https://github.com/sigven/vcf2tsvpy) and merged into one genome-wide file.

## gwRVIS

gwRVIS score were downloaded from https://az.app.box.com/v/jarvis-gwrvis-scores/folder/159239740414 and merged into one genome-wide file.

## Zoonomia

Mammalian PhyloP conservation scores were downloaded from https://hgdownload.cse.ucsc.edu/goldenpath/hg38/cactus241way/hg38.cactus241way.phyloP.bw, the primate specific PhyloP scores as well as 241-mammals runs of contiguous constraint (RoCCs) and ultra conserved elements (UCEs) were downloaded from https://cgl.gi.ucsc.edu/data/cactus/zoonomia-2021-track-hub/hg38/. BigWig files were converted to bigbed using bigWigToBedGraph (https://anaconda.org/bioconda/ucsc-bigwigtobedgraph).

# Supplementary Note S5: Training new CADD models

Training of CADD models (19) and annotation of the training data set (13, 19) were carried out as last described for CADD v1.6 (1). Briefly, CADD training data sets are unchanged from previous versions (for GRCh37 since CADD v1.3 and for GRCh38 since CADD v1.4). Proxy-neutral variants of the training data set are derived from human-derived fixed changed and 1000 Genome Project SNV and InDel variants with an allele frequency ≥ 95% that are absent in the inferred genome sequence of the human-ape ancestor, as identified from Ensembl EPO primate alignments (20) (using the respective GRCh37 or GRCh38 genome builds). Proxy-deleterious variants were then simulated using the sequence composition of the proxy-neutral variants (19), accommodating a local adjustment of mutation rates, specific substitution patterns and asymmetric CpG-specific mutation rates. The size of the simulated data was adjusted to the benign set so that the final training set is balanced in the number of proxy-benign and proxy-deleterious variants. The entire training data sets can be downloaded from https://cadd.gs.washington.edu/training or https://cadd.bihealth.org/training. See also main text chapter “Unique characteristics of the CADD framework” for more information on the generation of the training data set and model training. We trained logistic regression models

employing all features used in CADD v1.6 and additional features presented in this manuscript (c.f. Supplementary Table S1). Our final CADD v1.7 model uses 1213 features derived from more than 100 annotations. CADD trains a logistic model splitting this dataset into a training (99%) and test set (1%). Model parameters fitted after 13 training iterations using python's *scikit-learn* library are used as the final model and evaluation after each iteration is performed on the hold-out test set. The final model is then evaluated on external validation sets and benchmarked. Generation of test data sets used for model evaluation is explained in Supplementary Note S1.

# Supplementary Note S6: Performance metrics

We provide all variants used for testing our models as VCF files (see Supplementary Note S1). Note that for calculating CADD scores including the new features, we have in some cases excluded a small number of variants from the validation data sets when they are annotated as part of two or more genes by Ensembl VEP and in each gene with an effect of similar severity (e.g. missense vs splice change). In such cases, our internal workflow chooses one of these genes and its corresponding annotations randomly, potentially missing the specific gene for which the consequence annotation would fit the required benchmark. A small number of variants is than lost as we have explicitly excluded variants for which the CADD score does not depend on the respective new feature. E.g., in the Inframe InDel data set provided by Cannon et al. (11), 2088 of 2224 benign and 1665 of 1740 pathogenic variants remained. In the ClinVar **+ SNV missense dataset 7885 of 8033 benign and 4125 of 4231 variants remained. We do not expect these losses to affect our overall results.

## Spearman and Pearson correlations

Spearman or Pearson correlations were calculated with *python*'s *pandas* library. Corresponding uncertainties were calculated using 1,000 bootstrap iterations and sampling 80 % of the data. The resulting mean and standard deviation were used to estimate the mean value and its error (standard deviation). Note that for Spearman rho correlations of CADD scores and DMS scores of all variants (c.f. Fig. 2a, most right bars), we used the largest annotated DMS effect per variant, if a variant was included in multiple experiments in the ProteinGym data.

## ROC curves and AUROC values

True positive rates, true negative rates, and AUROC values were calculating using *pyhon*'s *scikit-learn* library. Mean and standard deviations of 1000 bootstrap iterations using 80% of the data were employed to estimate AUROC values and their uncertainties. Plotting of corresponding ROC curves was carried out by considering the entire and non-sampled data sets. For plotting ROCs of ESM missense and frameshift and stop gain scores, the scores were multiplied with -1 as they were negatively correlated with the pathogenic class and the *scikit-learn metrics.roc_curve* function expects a positive correlation for calculation of confusion matrices. Area under the curve values were calculated using *scikit-learn metrics.auc* function. For plotting the APARENT2 score on its own, we used its absolute value.

## Precision recall curves and average precision scores (APSs)

Precision values, recall values, and APS values were calculated using *python*'s *scikit-learn* library. Mean and standard deviations of 1000 bootstrap iterations using 80% of the data were employed to estimate APS values and their uncertainties. Plotting of corresponding precision recall curves was carried out by considering the entire and non-sampled data sets. For plotting precision recall curves and calculation of APS values with the benign class as positive class,

CADD scores were multiplied by -1 as they negatively correlated with the benign class and the *scikit-learn* functions *metrics.precision_recall_curve* and *metrics.average_precision_score* expect a positive correlation with the "positive class" for calculation of confusion matrices. For plotting precision recall curves of ESM scores for frameshift and stop gains and calculation of corresponding APSs, where the pathogenic class is used as positive class, ESM scores were multiplied by -1 to achieve a positive correlation. For plotting the APARENT2 score on its own, we used its absolute value.

# References


1. Rentzsch,P., Schubach,M., Shendure,J. and Kircher,M. (2021) CADD-Splice-improving genome-wide variant effect prediction using deep learning-derived splice scores. *Genome Med.*, **13**, 31.
2. Meier,J., Rao,R., Verkuil,R., Liu,J., Sercu,T. and Rives,A. (2021) Language models enable zero-shot prediction of the effects of mutations on protein function. In *Advances in Neural Information Processing Systems*. Curran Associates, Inc., Vol. 34, pp. 29287–29303.
3. Linder,J., Koplik,S.E., Kundaje,A. and Seelig,G. (2022) Deciphering the impact of genetic variation on human polyadenylation using APARENT2. *Genome Biol.*, **23**, 232.
4. Sullivan,P.F., Meadows,J.R.S., Gazal,S., Phan,B.N., Li,X., Genereux,D.P., Dong,M.X., Bianchi,M., Andrews,G., Sakthikumar,S., *et al.* (2023) Leveraging base-pair mammalian constraint to understand genetic variation and human disease. *Science*, **380**, eabn2937.
5. Seplyarskiy,V., Daniel J. Lee, Evan M. Koch, Joshua S. Lichtman, Harding H. Luan, and Shamil R. Sunyaev (2022) A mutation rate model at the basepair resolution identifies the mutagenic effect of Polymerase III transcription. *bioRxiv*, doi: https://doi.org/10.1101/2022.08.20.504670, 20 August 2022, pre-print: not peer-reviewed.
6. Vitsios,D., Dhindsa,R.S., Middleton,L., Gussow,A.B. and Petrovski,S. (2021) Prioritizing non-coding regions based on human genomic constraint and sequence context with deep learning. *Nat. Commun.*, **12**, 1504.
7. Bergstra,J., Bardenet,R., Bengio,Y. and Kégl,B. (2011) Algorithms for Hyper-Parameter Optimization. In. Neural Information Processing Systems Foundation, Vol. 24.
8. Bergstra,J., Yamins,D. and Cox,D. (2013) Making a Science of Model Search: Hyperparameter Optimization in Hundreds of Dimensions for Vision Architectures. In *International Conference on Machine Learning*. PMLR, pp. 115–123.
9. Notin,P., Dias,M., Frazer,J., Hurtado,J.M., Gomez,A.N., Marks,D. and Gal,Y. (2022) Tranception: Protein Fitness Prediction with Autoregressive Transformers and Inference-time Retrieval. In *Proceedings of the 39th International Conference on Machine Learning*. PMLR, pp. 16990–17017.
10. UniProt Consortium (2023) UniProt: the Universal Protein Knowledgebase in 2023. *Nucleic Acids Res.*, **51**, D523–D531.
11. Cannon,S., Williams,M., Gunning,A.C. and Wright,C.F. (2023) Evaluation of in silico pathogenicity prediction tools for the classification of small in-frame indels. *BMC Med. Genomics*, **16**, 36.
12. Landrum,M.J., Lee,J.M., Riley,G.R., Jang,W., Rubinstein,W.S., Church,D.M. and Maglott,D.R. (2014) ClinVar: public archive of relationships among sequence variation and human phenotype. *Nucleic Acids Res.*, **42**, D980–D985.
13. Rentzsch,P., Witten,D., Cooper,G.M., Shendure,J. and Kircher,M. (2019) CADD: predicting the deleteriousness of variants throughout the human genome. *Nucleic Acids Res.*, **47**, D886–D894.
14. The 1000 Genomes Project Consortium, Abecasis,G.R., Auton,A., Brooks,L.D., DePristo,M.A., Durbin,R.M., Handsaker,R.E., Kang,H.M., Marth,G.T. and McVean,G.A. (2012) An integrated map of genetic variation from 1,092 human genomes. *Nature*, **491**, 56–65.
15. Kircher,M., Xiong,C., Martin,B., Schubach,M., Inoue,F., Bell,R.J.A., Costello,J.F., Shendure,J. and Ahituv,N. (2019) Saturation mutagenesis of twenty disease-associated regulatory elements at single base-pair resolution. *Nat. Commun.*, **10**, 1–15.
16. McLaren,W., Gil,L., Hunt,S.E., Riat,H.S., Ritchie,G.R.S., Thormann,A., Flicek,P. and Cunningham,F. (2016) The Ensembl Variant Effect Predictor. *Genome Biol.*, **17**, 122.

17. Quinlan,A.R. and Hall,I.M. (2010) BEDTools: a flexible suite of utilities for comparing genomic features. *Bioinformatics*, **26**, 841–842.

18. Krützfeldt,L.-M., Schubach,M. and Kircher,M. (2020) The impact of different negative training data on regulatory sequence predictions. *PLOS ONE*, **15**, e0237412.

19. Kircher,M., Witten,D.M., Jain,P., O'Roak,B.J., Cooper,G.M. and Shendure,J. (2014) A general framework for estimating the relative pathogenicity of human genetic variants. *Nat. Genet.*, **46**, 310–315.

20. Herrero,J., Muffato,M., Beal,K., Fitzgerald,S., Gordon,L., Pignatelli,M., Vilella,A.J., Searle,S.M.J., Amode,R., Brent,S., *et al.* (2016) Ensembl comparative genomics resources. *Database J. Biol. Databases Curation*, **2016**, bav096.
